# CRISPR/Cas9-mediated knockout of *NYC1* gene enhances chlorophyll retention and reduces tillering in *Zoysia matrella* (L.) Merrill

**DOI:** 10.1007/s00299-023-03130-6

**Published:** 2024-02-02

**Authors:** Hwan May Ng, Takahiro Gondo, Hidenori Tanaka, Ryo Akashi

**Affiliations:** 1 Interdisciplinary Graduate School of Agriculture and Engineering, University of Miyazaki, Miyazaki, Japan; 2 Frontier Science Research Center, University of Miyazaki, Miyazaki, Japan; 3 Faculty of Agriculture, University of Miyazaki, Miyazaki, Japan; 4 University of Miyazaki, Miyazaki, Japan

**Keywords:** Zoysiagrass, Genome editing, CRISPR/Cas9, Stay-green phenotype, *NYC1* gene, Chlorophyll content

## Abstract

**Key Message:**

**Genome editing by CRISPR/Cas9 can be applied to** ***Z. matrella*** **‘Wakaba’, and knockout mutants of** ***ZmNYC1*** **gene exhibited stay-green phenotype and reduced tillering.**

**Abstract:**

*Zoysia matrella* is a widely used C4 warm-season turfgrass for landscaping, golf courses, and sports fields. Here, we used the CRISPR/Cas9 system to target the *Non-Yellow Coloring1* (*ZmNYC1*) gene in the highly heterozygous allotetraploid *Z. matrella* ‘Wakaba’, aiming to generate a novel stay-green variety. Of 441 *Agrobacterium*-infected calli, 22 (5.0%) were transformed, and 14 of these (63.6%) showed targeted mutations through cleaved amplified polymorphic sequences analysis. Sequencing analysis revealed mutations mostly consisting of 1 or 2 bp indels, occurring 2 to 4 bp upstream of the PAM sequence. Regenerated plants exhibited five *ZmNYC1* target locus genotypes, including homozygous mutants with a complete knockout of all four alleles in the T0 generation. Under dark treatment, *ZmNYC1*-mutated plants displayed suppressed chlorophyll *b* (Chl *b*) degradation, leading to higher chlorophyll content and Chl *b*, with a lower chlorophyll *a*/chlorophyll *b* ratio compared to the wild type (WT). However, the *ZmNYC1* mutation also inhibited plant growth in homozygous mutant genotypes, exhibiting reduced tillering compared to WT. Additionally, during winter simulation, mutant with a complete knockout retained greenness longer than the WT. This is the first successful use of CRISPR/Cas9 genome editing in zoysiagrass. The mutants of the *ZmNYC1* gene would serve as valuable breeding material for developing improved zoysiagrass varieties that can maintain their green color for longer periods, even during winter dormancy.

**Supplementary Information:**

The online version contains supplementary material available at 10.1007/s00299-023-03130-6.

## Introduction

Zoysiagrass, a common C4 warm-season turfgrass, is native to the western Pacific Rim and Indian Ocean regions (Loch et al. 2017). It belongs to the *Zoysia* genus, which comprises 11 species. These species are allotetraploids exhibiting an AABB genome type, sharing an identical chromosome count of 2n = 4x = 40, and there are no chromosomal barriers to interspecific crossing (Forbes 1952; Tsuruta et al. 2011). Among these species, *Zoysia japonica* Steudel, *Z. matrella* (L.) Merrill, *Z. pacifica* (Goudswaard) M. Hotta are three commercially important species in Japan. Genetic variation and population structure analysis indicated that *Z. matrella* is conceivable to be an interspecific hybrid originating from a cross between *Z. japonica* and *Z. pacifica* (Kimball et al. 2013; Tanaka et al. 2016b). In addition, *Z. matrella* exhibits intermediate morphology between *Z. japonica* and *Z. pacifica* and is valued for its superior turf quality and desirable traits (Hashiguchi et al. 2006; Kunwanlee et al. 2018). The turf of *Z. matrella* is dense and fine-textured, enhancing its visual appeal and providing a pleasant walking surface, making it suitable for extensive use in landscaping, golf courses, parks, and sports fields.

The demand for year-round green turf is experiencing significant growth, particularly in sports turf industries. Similar to all perennial warm-season C4 grasses, *Z. matrella* undergoes a dormant phase in its annual life cycle as a protective mechanism during the winter months. Zoysiagrass lawns exhibit a brownish-white appearance by chlorophyll degradation, leading to concomitant reductions in aesthetics, function, and recreational quality over 5 to 6 months in winter, depending on the genotype (Pompeiano et al. 2015). To mitigate the challenge of dormant turf, turf colorant or winter overseeding are common practices to provide a yearlong green turf (Braun et al. 2015). However, these procedures cause substantial investments in terms of both effort and financial resources. Therefore, the development of improved cultivars with stay-green traits is desirable for increasing the utilization value and sustainable maintenance of zoysiagrass.

Leaf senescence is primarily characterized by the degradation of chlorophyll, and this process is regulated by a range of chlorophyll catabolic enzymes (Hörtensteiner 2013) and senescence-associated genes (Balazadeh et al. 2008). The chlorophyll catabolic enzyme-encoding genes identified so far in higher plants are *chlorophyllide a oxygenase* (*CAO*), *Non-Yellow Coloring1* (*NYC1*), *NYC1-Like* (*NOL*), 7-hydroxymethyl Chl *a* reductase (*HCAR*), *Staygreen* (*SGR*), *Pheophytinase* (*PPH*), *Chlorophyllase* (*CLH*), *Pheide a oxygenase* (*PAO*), and *Red chlorophyll catabolite reductase* (*RCCR*) (Hu et al. 2021). Among these genes, *NYC1* encodes a chloroplast-localized short-chain dehydrogenase/reductase and acts as a chlorophyll *b* (Chl *b*) reductase responsible for catalyzing the degradation of Chl *b* (Kusaba et al. 2007; Sato et al. 2009). *NYC1* also plays a crucial role in the degradation of the light-harvesting complex II (LHCII) and the thylakoid membrane (Kusaba et al. 2007; Horie et al. 2009). Mutants of the *NYC1* gene in *Arabidopsis* and rice resulted in delayed leaf yellowing and the development of a stay-green phenotype due to the disrupted degradation of Chl *b* (Kusaba et al. 2007; Sato et al. 2009, 2018; Jibran et al. 2015). A recent study demonstrated the significant involvement of the *Z. japonica NYC1* (*ZjNYC1*) gene in accelerating chlorophyll degradation and promoting senescence through its overexpression in *Arabidopsis thaliana* (Teng et al. 2021).

Conventional cross-breeding has been the primary method for breeding new zoysiagrass varieties, however, the absence of stay-green traits in its genetic resources makes the development of such varieties impossible using this method. Targeted mutagenesis by genome editing has emerged as an effective tool that enables the generation of desired mutations in specific target genes across various crop species. Therefore, this new breeding technology provides a valuable alternative to address this limitation. CRISPR/Cas9 system has become the preferred tool for genome editing, contributing to great success in targeted mutagenesis in cereal crops such as rice (*Oryza sativa*), wheat (*Triticum aestivum*), and maize (*Zea mays*), as well as forage grasses like perennial ryegrass (*Lolium perenne*) and switchgrass (*Panicum virgatum* L.) (Zhang et al. 2019; Bao et al. 2022). Although significant progress has been made in Poaceae species using this technique, as far as the authors are aware, there have been no reports of CRISPR/Cas9 genome editing in zoysiagrass.

In this study, we performed genome editing by targeting the *Z. matrella* ‘Wakaba’ *NYC1* (*ZmNYC1*) loci using the *Agrobacterium*-mediated CRISPR/Cas9 system. Through this approach, we achieved efficient targeted mutagenesis and obtained knockout mutants of both sub-genomes of the *ZmNYC1* gene in the T0 generation. Under dark-induced and winter simulation treatments, these mutants exhibited a longer stay-green phenotype than the wild type (WT). The successful genome editing in *Z. matrella* in this study will not only contribute to the advancement of genetic breeding techniques in zoysiagrass, but the resulting stay-green mutant lines will also serve as novel breeding material for the development of valuable varieties.

## Material and methods

### Plant materials and embryogenic callus culture conditions

The plant material was *Z*. *matrella* ‘Wakaba’, grown in the greenhouse of the University of Miyazaki. Embryogenic callus was induced from the stolon node and yellowish and compact callus with dense poly-embryogenic clusters on the surface were subcultured every 2 weeks. These highly regenerative embryogenic calli were cultured on MS medium supplemented with 2 mg/L 2,4-D, 0.1 mg/L BAP, and 5 μM CuSO_4_ (MS-DBC medium) at 27 °C in the dark, according to our established procedure (Ng et al. 2023).

### Guide RNA (gRNA) design and vector construction

**Fig. 1:**
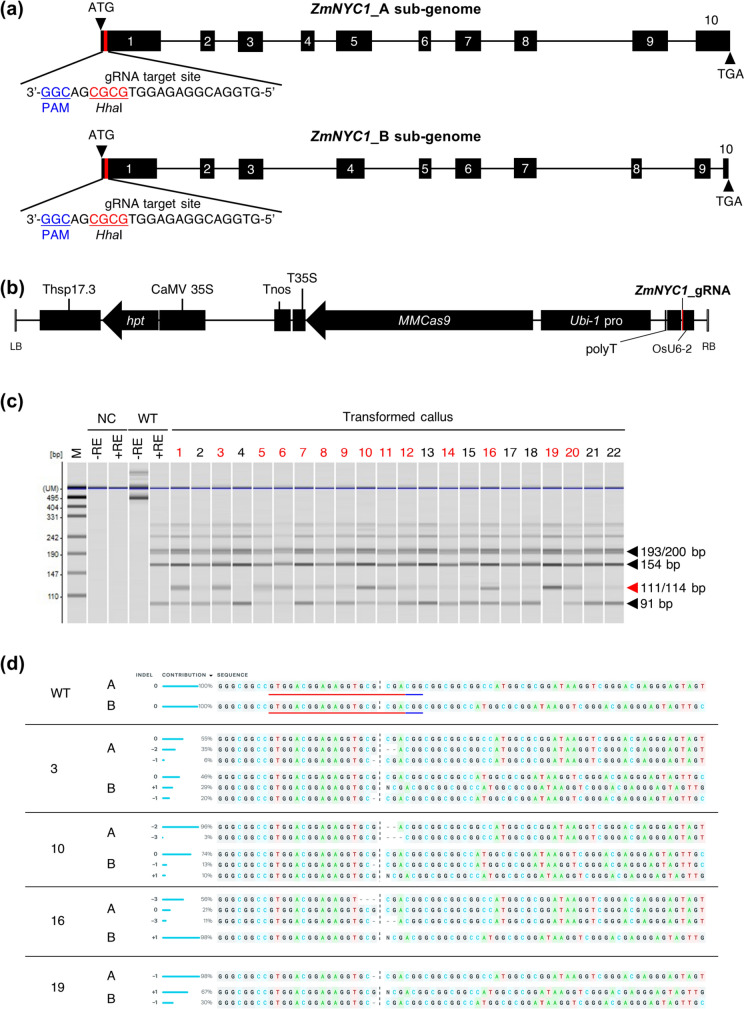
CRISPR/Cas9-mediated mutagenesis of *ZmNYC1* and targeted mutation confirmation in transformed calli:** a** Exons of the heterozygous *Z. matrella* ‘Wakaba’ *NYC1* (*ZmNYC1*_A and *ZmNYC1*_B) gene are shown as black squares, and introns are shown as black lines. The gRNA is indicated by red lines, targeting exon 1 of the *ZmNYC1* loci using the CRISPR/Cas9 system. The protospacer-adjacent motif sequence (PAM) is shown in blue, and the *Hha*I restriction site is shown in red. **b** The CRISPR/Cas9 vector pZH_OsU6gRNA_PubiMMCas9-*ZmNYC1* used for the transformation, containing expression cassettes for one gRNA (OsU6-2pro: *ZmNYC1*_gRNA (red line): polyT), Cas9 (Ubi-1pro: *Cas9*: T35STnos), and hygromycin phosphotransferase (*hpt*) gene (CaMV35Spro: *hpt*: Thsp17.3). **c** CAPS analysis of the *ZmNYC1* mutation site using A and B sub-genomes-common primer (*ZmNYC1_*AB). Transformed calli with mutations are indicated in red. Amplicons were digested with the *Hha*I restriction enzyme at the Cas9 cleavage site to detect wild type (WT) (digested, black arrowheads) and mutant (undigested, red arrowhead) plants. During targeted mutation, the *Hha*I recognition site located between 23 and 91 bp in the A sub-genome and between 20 and 91 bp in the B sub-genome cannot be digested, resulting in fragment sizes of 114 bp and 111 bp, respectively (see also Fig. S1). Bands of 23 and 20 bp have been omitted from the gel image as they were too small to be visible and insignificant to the result. *M* pUC19 DNA/MspI (HpaII) marker, *NC* negative control, − *RE* without restriction enzyme, + *RE* with restriction enzyme. **d** Analysis of the gRNA-targeted *ZmNYC1* region displaying the generated mutation spectrum. Data were generated by the Inference of CRISPR Edits (ICE) v2 bioinformatic tool on Sanger sequencing of A and B sub-genomes

For the design of gRNA, the heterozygous sequences of the *Z. matrella NYC1* (*ZmNYC1*) gene (Zmw_sc04443.1.g00090.1.am.mkhc, Zmw_sc02351.1.g00130.1.sm.mkhc) were obtained from the Zoysia Genome Database (http://zoysia.kazusa.or.jp/) (Tanaka et al. 2016a). The gRNA on-target activity was predicted using the WU-CRISPR tools (Wong et al. 2015). To ensure the suitability of the gRNA and the Cas9 nuclease scaffold, the predicted secondary structures of the single-stranded RNA, including the target site and the Cas9 scaffold, were assessed using the RNAfold web server (http://rna.tbi.univie.ac.at/cgi-bin/RNAWebSuite/RNAfold.cgi). One gRNA was designed to simultaneously target both exon 1 of Zmw_sc04443.1.g00090.1.am.mkhc (A sub-genome) and Zmw_sc02351.1.g00130.1.sm.mkhc (B sub-genome), designated as *ZmNYC1*: 5′-GTGGACGGAGAGGTGCGCGA-3′ (Fig. 1a). This design was based on the presence of the PAM sequence, which both sub-genomes share. Additionally, the recognition sequence of the common restriction enzyme *Hha*I (underlined in the gRNA targets in Fig. 1a) was included for cleaved amplified polymorphic sequences (CAPS) analysis.

The CRISPR/Cas9 vector pZH_OsU6gRNA_PubiMMCas9-*ZmNYC1* was constructed according to previous reports (Mikami et al. 2015; Abe et al. 2020) (Fig. 1b). In brief, the complementary DNA for the gRNA target was cloned into a *Bbs*I-digested site in pU6gRNA-oligo, and the expression cassettes of targets containing gRNA scaffold sequences under the control of the OsU6 promoter were excised with *Pac*I and *Asc*I and then cloned into destination vector pZH_gYSA_PubiMMCas9 harboring the maize ubiquitin promoter. The CRISPR/Cas9 all-in-one vector was transformed into *Agrobacterium tumefaciens* strain EHA105 via heat-shock protocol (Hofgen and Willmitzer 1988). The resulting *Agrobacterium* strain was used for *Z. matrella* transformation.

### Agrobacterium-mediated transformation of Z. matrella ‘Wakaba’

*Agrobacterium*-mediated transformation of *Z. matrella* ‘Wakaba’ was conducted as described in our previous report (Ng et al. 2023), with slight modifications. Calli were co-cultivated with the *A. tumefaciens* for 5 days at 25 °C in the dark. Subsequently, calli were cultured on an MS-DBC medium supplemented with 50 mg/L meropenem and 75 mg/L hygromycin (MS-SE) at 31 °C in the dark. Subculture was performed every 2 weeks under the same conditions until the formation of hygromycin-resistant calli can be identified after 60–80 days. Subcultures of the resistant calli were continuously maintained to promote proliferation for subsequent plant regeneration. The resistant calli were then transferred to a hormone-free MS medium (MS-FSE) containing 50 mg/L meropenem and 50 mg/L hygromycin for plant regeneration and cultured at 27 °C under a 16 h light/8 h dark photoperiod for 2 months. Regenerated plants were separated into single tillers, treated as independent plants and transferred to the hormone-free half-strength MS major salts medium (^1^/_2_ MS) for rooting. After 2–3 weeks, rooted plants were transferred to pots containing a mixture of soil and vermiculite (10:3) and grown in a growth chamber at 28 °C under a 16 h light/8 h dark photoperiod. Plants were watered every other day and fertilized biweekly with 500-fold diluted Hyponex fertilizer (Hyponex Japan, Osaka, Japan).

### Detection of targeted mutations by CAPS and sequence analysis

Genomic DNA from resistant callus and regenerated plants were extracted using the FavorPrep™ Plant Genomic DNA Extraction mini kit (Favorgen Biotech Corp., Taiwan) and the Kaneka Easy DNA Extraction Kit version 2 (Kaneka, Tokyo, Japan), respectively. Each round of PCR was conducted in a reaction mixture (10 μL) containing dNTP (0.4 mM of each), 1 × PCR Buffer for KOD FX, primers (300 nM of each), KOD FX (1.0 U; TOYOBO, Japan), and genomic DNA. The genomic DNA used was 1.0 μL of 10 ng for the resistant callus and tenfold dilution for leaf tissue. The reaction mixture was denatured for 2 min at 94 °C in a thermocycler, followed by either 30 cycles for resistant callus or 40 cycles for leaf tissue amplification (98 °C for 10 s, 68 °C for 30 s). The primer sets for the conserved *ZmNYC1* genes, either specific or common to both A and B alleles, are described in Table S1. PCR products were subjected to digestion with the restriction enzyme *Hha*I and analyzed using microchip electrophoresis (MultiNA, Shimadzu). PCR products amplified with sub-genome-specific primers were cleaned up for sequencing analysis using ExoSAP-IT Express (Thermo Fisher Scientific) and subsequently sequenced using either ABI 3130 or ABI 3500xL sequencer (Applied Biosystems). The resulting chromatograms were analyzed for mutation patterns using the Inference of CRISPR Edits (ICE) v2 bioinformatic tool (Conant et al. 2022), with the chromatograms of the wild type used as the control trace. The Knock-out Score (KO score) was calculated as the proportion of cells with frameshift or indels that could contribute to a functional knockout of the target gene.

### Analysis of chlorophyll content and photosynthetic efficiency after dark treatment

The T0 mutants were categorized into AaBb, AAbb, Aabb, and aabb genotypes based on the mutation patterns of the target loci of the A and B alleles. After being transferred to soil for 3 months, both the WT and transgenic plant lines were subjected to dark treatment. Three technical replicates were conducted for each T0 mutant, and the means of mutant genotypes were calculated based on these mutants, categorized by their genotype mutation patterns for analysis. Dark incubation was carried out with the full-length second leaf of each plant line. The detached leaves were placed between Prowipes moistened with sterile deionized water, sealed in a zipper storage bag, and incubated at 28 °C in the dark for up to 8 days. Photos of dark-induced leaves were taken on Days 0, 4, and 8, respectively (Fig. S1). Treated leaves were used for chlorophyll content assessment on Days 0 and 8. The fresh weight of the treated leaves was measured before chlorophyll extraction. Leaf tissues were frozen in liquid nitrogen and disrupted using a TissueLyser II (Qiagen) with 7 mm zirconia balls for 30 s at 30 Hz. Chlorophyll was extracted using 80% acetone, and chlorophyll concentrations were determined according to Porra et al. (1989). The Fv/Fm ratio of the dark-induced leaf was measured on Days 0, 4, and 8 using a FluorPen FP 110 fluorometer (Photon Systems Instruments). The measurements were taken at three positions (top, middle, and bottom) after a 15-min dark adaptation, following the manufacturer's instructions. The Fv/Fm ratio was calculated by averaging the values from these measurements. Data were analyzed through a one-way ANOVA with a post hoc LSD test. *P* < 0.05 were considered statistically significant. All data are presented as the mean ± SD (*n* ≥ 3).

### Plant phenotype and measurement of tiller number

After 3 months of growth in the growth chamber, the transgenic plants were transplanted into 10 cm in diameter pot and transferred to a GM greenhouse. Photos were taken to observe the growth and size differences among the different mutant genotype plants after 42 days of clipping the plants to a height of 15 mm. Tillers were counted approximately seven months after the transfer. Data analysis was conducted as described in the dark treatment analysis.

### Analysis of chlorophyll content after simulated winter condition

After being transplanted into the soil for 3 months, both the WT and mutant line 19–1 were exposed to cold and short-day treatment simulating winter conditions. Four pots were used as biological repeats for chlorophyll content measurements (*n* = 4). The plants were incubated in a growth chamber with a constant temperature of 5 °C and a short-day photoperiod of 8 h light/16 h dark (0, 14, 28, and 42 days). Photos of the treated plants were taken at each time point. Chlorophyll extraction was conducted on the second leaf at each time point following the same procedure as described in the dark treatment analysis.

## Results

### Identification of transformed callus with targeted mutations in ZmNYC1

To perform genome editing of the *ZmNYC1* loci, a 20-bp gRNA sequence was designed to target the conserved region among the two sub-genomes (*ZmNYC1*_A and *ZmNYC1*_B) in exon 1, which serves as a target of CRISPR/Cas9 (Fig. 1a). An all-in-one plasmid vector carrying both the gRNA and the Cas9 expression cassette was introduced into the embryogenic callus through *Agrobacterium*-mediated transformation system (Ng et al. 2023) in *Z. matrella* ‘Wakaba’ (Fig. 1b). Of 441 *Agrobacterium*-infected calli, 22 transformed calli were obtained, resulting in a transformation efficiency of 5.0% (Table 1).

**Table 1:** Summary of *Agrobacterium*-mediated transformation and targeted mutations of *ZmNYC1* gene in *Z. matrella* ‘Wakaba’

Experiment	*Agrobacterium-*infected callus	Transformed callus (%)	Callus with mutation^a^	Mutation efficiency (%)^b^
1	84	6 (7.1)	4	66.7
2	189	13 (6.9)	7	53.9
3	168	3 (1.8)	3	100.0
Total	441	22 (5.0)	14	63.6

^a^Mutations identified by CAPS analysis

^b^The number of callus with mutation divided by the number of transformed callus

To detect mutations in the *ZmNYC1* gene, genomic DNA was extracted from 22 transformed calli, and PCR was performed using A and B sub-genomes-common primer (Table S1). As the CRISPR/Cas9 cleavage site was designed to contain a restriction enzyme recognition site (*Hha*I) on the target gene, mutated sequences were expected to remain undigested, leading to a detectable band at around 111/114-bp (Fig. 1c, Fig. S1). Targeted mutations were identified in 14 of the 22 transformed calli, indicating a mutation efficiency of 63.6%. Among these, 13 calli showed heterozygous bands, and one callus (line 19) showed a homozygous band, indicating mutations in all four target *ZmNYC1* loci of both A and B sub-genomes.

PCR products amplified using the sub-genome-specific primers from all transformed calli were subjected to direct sequencing, and Sanger sequencing chromatograms were then analyzed using the Inference of CRISPR Edits (ICE) v2 bioinformatic tool (Conant et al. 2022). Mutations with an indel ratio greater than or equal to 5% as identified by ICE analysis (Table S2), are summarized in Table 2. Based on this criterion, 14 of the 22 transformed calli exhibited targeted mutations. The observed homozygous and heterozygous mutant band patterns at the *ZmNYC1* locus in the CAPS analysis (Fig. 1c) were also consistent with the results of ICE analysis. Among the 14 mutant callus lines, mutations in A sub-genome alleles included -7, -3, -2, -1, and + 1 bp, while mutations in B sub-genome alleles comprised -6, -1, and + 1, occurring 2 to 4 bp upstream of the PAM sequence (Table S2). Six and eight lines had KO scores of 30% or higher for targeted mutations in the A and B sub-genomes, respectively. The homozygous mutated line 19 showed a KO score of 98% with a 1-bp deletion in A sub-genome alleles, while B sub-genome alleles had a KO score of 97% with a 30% indel ratio of 1-bp deletion and a 67% 1-bp insertion.

**Table 2:** Summary of mutations at target *ZmNYC1* loci in genome-edited calli

Callus line	A allele		B allele	
	Indel (%)^a^	Knock-out score (%)^b^	Indel (%)^a^	Knock-out score (%)^b^
1	− 2 (5)/− 1 (16)/0 (73)	21	− 1 (42)/0 (13)/ + 1 (41)	83
2	0 (100)	0	0 (95)	0
3	− 2 (35)/− 1 (6)/0 (55)	41	− 1 (20)/0 (46)/ + 1 (29)	49
4	0 (100)	0	0 (100)	0
5	− 7 (6)/0 (48)/ + 1 (40)	46	− 2 (30)/− 1 (10)/0 (38)/ + 1 (18)	58
6	− 1 (40)/0 (33)/ + 1 (19)	59	− 1 (25)/0 (7)/ + 1 (65)	90
7	0 (99)	0	0 (71)/ + 1 (27)	27
8	− 1 (30)/0 (68)	30	− 1 (5)/0 (71)/ + 1 (22)	27
9	0 (92)/ + 1 (5)	5	0 (92)/ + 1 (6)	6
10	− 2 (96)	96	− 1 (13)/0 (74)/ + 1 (10)	23
11	0 (95)	0	+ 1 (98)	98
12	0 (100)	0	0 (88)/ + 1 (9)	9
13	0 (100)	0	0 (100)	0
14	0 (92)	0	− 6 (89)/0 (10)	0
15	0 (100)	0	0 (94)	0
16	− 3 (56)/− 3 (11)/0 (21)	0	+ 1 (98)	98
17	0 (99)	0	0 (94)	0
18	0 (100)	0	0 (99)	0
19	− 1 (98)	98	− 1 (30)/ + 1 (67)	97
20	− 2 (5)/0 (94)	5	0 (8)/ + 1 (89)	89
21	0 (100)	0	0 (99)	0
22	0 (100)	0	0 (100)	0

^a^Indels were predicted by the ICE Synthego tool. Indels greater than or equal to 5% were considered mutations but less than 5% were not accepted as mutations due to some inherent noise in the Sanger sequencing. Indels indicate −; deletion, + ; insertion, and the number of nucleotides

^b^Knock-out score represents the proportion of cells with frameshift or indels that could contribute to a functional knockout of the target gene

### Identification and characterization of the targeted mutations in regenerated plants

**Fig. 2:**
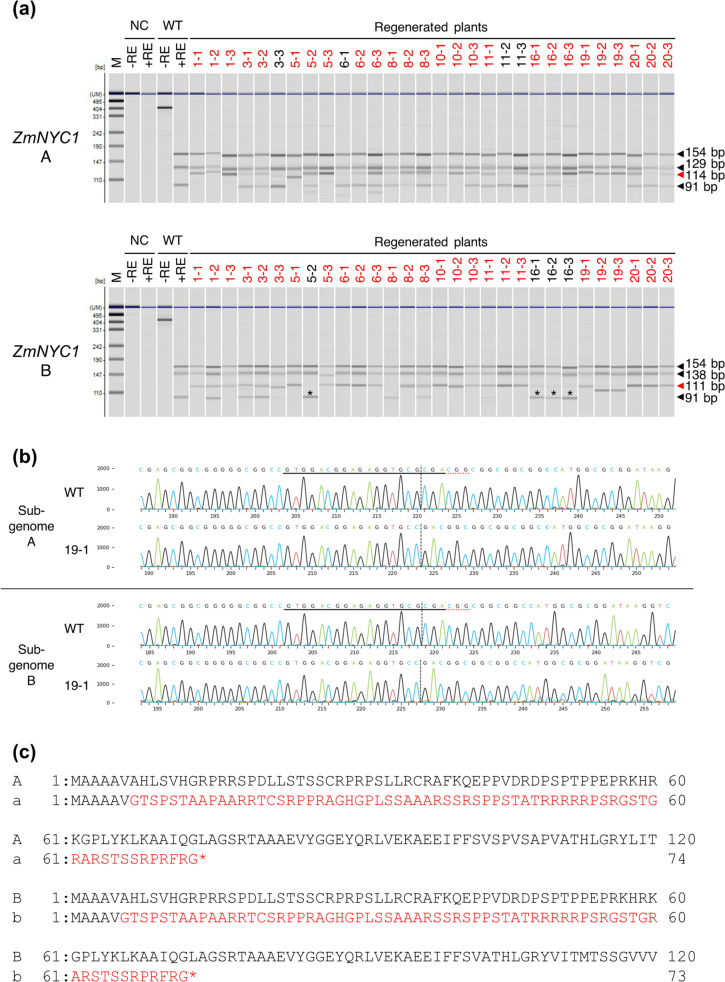
Mutation of *ZmNYC1* in the A and B sub-genomes of T0 plants. **a** CAPS analysis of the A and B sub-genomes. T0 plants with mutations are indicated in red. Amplicons were digested with the *Hha*I restriction enzyme at the Cas9 cleavage site to detect wild type (WT) (digested, black arrowheads) and mutant (undigested, red arrowhead) plants. *M* pUC19 DNA/MspI (HpaII) marker, *NC* negative control, − *RE* without restriction enzyme, + *RE* with restriction enzyme. Asterisks indicate plant lines with mutations not detected with CAPS analysis due to no alteration to the coding sequences of the *Hha*I restriction enzyme (GCGC). **b** Sanger sequencing analysis showing genome editing in the *ZmNYC1* loci of mutant line 19–1. Black underline represents gRNA and red dotted lines represent PAM sequence. Vertical dotted lines indicate indel position. **c** Deduced amino acid sequences around the target sites. The sub-genomes A and B represent WT; a and b represent line 19–1. One base deletion causes a frameshift. The resulting amino acid sequences are shown below in red

Ten transformed calli with a high mutation frequency of either the A or B sub-genome, with a KO score over 30%, were selected for plant regeneration (Table 2). A total of 30 regenerated plants, three from each callus line, were subjected to CAPS analysis. Among these, 26 regenerated plants showed targeted mutations at either the A or B sub-genome, while 22 plants exhibited targeted mutation for both alleles simultaneously (Fig. 2a). Mutations with an indel ratio greater than 10% as identified by ICE analysis (Table S3), are summarized in Table 3. The indel threshold of 10% for ICE analysis was consistent with the mutation band pattern observed in the CAPS analysis except for plant lines 5–2, 16–1, 16–2, and 16–3 in the B sub-genome. This was due to a one-base cytosine (C) insertion at the 4th bp upstream of PAM in the B sub-genome of these lines, which did not alter the coding sequences of the *Hha*I restriction enzyme (GCGC) (Fig. 2a, Table S3).

**Table 3:** Summary of mutations at target *ZmNYC1* loci in genome-edited T0 plants

Line	Plant	Genotype^a^	A allele		B allele	
			Mutation type^b^	Indel (%)^c^	Mutation type ^b^	Indel (%)^c^
1	1	aabb	B	− 1 (78)/ + 1 (13)	H	− 1 (99)
	2	aaBb	C	− 4 (22)/− 4 (24)/− 1 (45)	M	0 (61)/ + 1 (33)
	3	aabb	B	− 2 (14)/− 1 (85)	B	− 1 (89)/ + 2 (10)
3	1	AaBb	M	− 1 (10)/0 (89)	M	0 (56)/ + 1 (39)
	2	AaBb	M	− 1 (42)/0 (55)	M	− 1 (16)/0 (73)
	3	AAbb	WT	0 (99)	H	− 2 (87)
5	1	aabb	H	− 7 (92)	H	+ 1 (99)
	2	Aabb	C	− 2 (53)/− 1 (17)/0 (21)	H	+ 1 (96)
	3	aabb	H	− 1 (99)	H	+ 2 (80)
6	1	AAbb	WT	0 (99)	H	+ 1 (99)
	2	Aabb	M	− 2 (16)/0 (82)	H	+ 1 (98)
	3	Aabb	M	− 2 (40)/0 (49)	H	+ 1 (98)
8	1	AaBb	M	− 1 (35)/0 (62)	M	− 1 (10)/0 (79)
	2	Aabb	C	− 2 (48)/− 1 (14)/0 (16)/ + 1 (17)	C	− 2 (22)/− 1 (33)/ + 1 (34)
	3	AaBb	M	0 (59)/ + 1 (19)	C	− 1 (40)/0 (38)/ + 1 (13)
10	1	Aabb	M	− 2 (29)/0 (59)	H	+ 1 (98)
	2	aabb	H	− 2 (99)	B	− 1 (49)/ + 1 (45)
	3	Aabb	M	− 1 (32)/0 (65)	H	+ 1 (98)
11	1	Aabb	M	− 2 (25)/0 (70)	B	+ 1 (86)/ + 3 (11)
	2	AAbb	WT	0 (92)	H	+ 1 (99)
	3	AAbb	WT	0 (91)	H	+ 1 (98)
16	1	aabb	B	− 3 (67)/− 2 (11)	H	+ 1 (95)
	2	Aabb	C	− 3 (19)/− 3 (29)/0 (42)	H	+ 1 (96)
	3	aabb	H	− 1 (97)	H	+ 1 (99)
19	1	aabb	H	− 1 (99)	H	− 1 (94)
	2	aabb	C	− 3 (10)/− 3 (12)/− 1 (74)	H	− 8 (100)
	3	aabb	H	− 3 (90)	H	− 8 (100)
20	1	Aabb	M	− 2 (30)/0 (53)	H	+ 1 (99)
	2	Aabb	M	− 1 (24)/0 (74)	H	+ 1 (98)
	3	Aabb	M	− 2 (24)/0 (63)	H	+ 1 (98)

^a^The genotype was categorized according to indel at the target *ZmNYC1* loci

^b^The mutation type of each sub-genome is shown as follows. *WT* wild type, *B* bi-allelic mutation, *M* monoallelic mutation, *C* chimeric mutation, *H* homoallelic mutation

^c^Indels were predicted by the ICE Synthego tool. Indels greater than or equal to 10% were considered mutations but less than 10% were not accepted as mutations due to some inherent noise in the Sanger sequencing. Indels indicate −, deletion, + , insertion and the number of nucleotides

The indel patterns of T0 transgenic mutants displayed multiple editing events, despite deriving from the same callus line (Tables 2 and 3). Some plants carried new mutations not previously observed in the initial callus lines, and chimeric plants with three different indels occurring in either sub-genome were also observed. The T0 plant lines exhibited five genotypes for the *ZmNYC1* target loci, AAbb, AaBb, Aabb, aaBb, and aabb, based on the mutation type indicated by the indel pattern of the A, B sub-genome. Chimeric mutation types were assumed to be the mutant genotypes. Ten of 30 regenerated plants showed the aabb genotype in the T0 generation (Table 3), especially in plants from line 19, where the KO rate was almost 100% in the callus mutation analysis, resulting in all regenerated plants exhibiting the aabb genotype. Plant line 19–1 was a complete homozygous mutant with a 1 bp deletion in both A and B sub-genomes, leading to a stop codon at the 74th codon in A sub-genome and the 73rd codon in B sub-genome due to frameshifts (Fig. 2c).

### Stay-green and plant phenotype of the different ZmNYC1 mutant genotypes

**Fig. 3:**
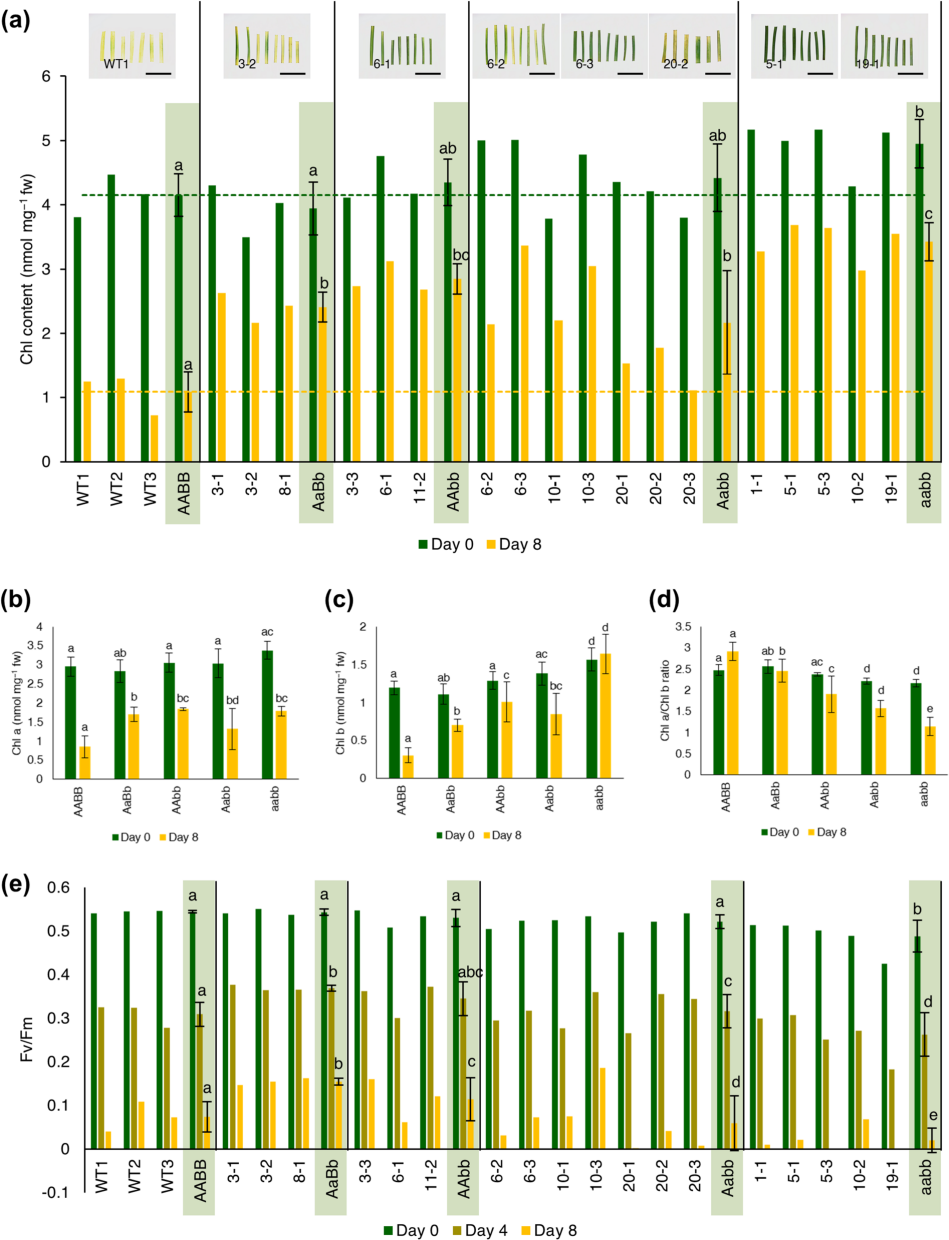
Characteristics of the stay-green phenotype during dark-induced senescence in different *ZmNYC1* mutant genotypes. **a** Chlorophyll content at Day 0 and Day 8 and the representative photos of mutant lines for each genotype compared with wild type (WT) at Day 8. Dotted lines represent the average chlorophyll content of the WT at Day 0 (green) and Day 8 (yellow). Scale bar: 1 cm. **b** Chlorophyll *a* content, **c** Chlorophyll *b* content and **d** Chlorophyll *a*/Chlorophyll *b* ratio for each genotype at Day 0 and Day 8. **e** Fv/Fm values at Day 0, Day 4, and Day 8. Values of each plant line are the mean of three technical replicates. Mean of genotypes are the mean of plant lines of the same genotype. Error bars represent the standard deviation (SD). Letters represent significant differences on each treatment day determined by one-way ANOVA with a post hoc LSD test (*P* < 0.05, *n* ≥ 3)

A total of 18 of the 30 mutants were utilized in the dark treatment experiment, excluding chimeric mutants and those lacking a frameshift mutation as identified through sequencing analysis. Based on the indels within the alleles of A and B sub-genomes, the mutants were categorized into genotype patterns: AaBb, AAbb, Aabb, and aabb. After 8 days of dark treatment, detached leaves of WT (AABB) turned yellow, while some mutated T0 plants remained green especially leaves of aabb mutant genotype (Fig. 3a and S2). This result was also evident in chlorophyll content, where the aabb mutant genotype exhibited significantly higher chlorophyll content than the WT even at Day 0 of treatment, and at Day 8, the chlorophyll content was approximately three times higher than that of the WT (Fig. 3a). Similarly, the other mutant genotypes also demonstrated significantly higher chlorophyll content compared to the WT at Day 8. While chlorophyll *a* (Chl *a*) was minimally affected by genotype variations (Fig. 3b), chlorophyll *b* (Chl *b*) was strongly affected by the mutant genotype (Fig. 3c). The aabb mutant genotype had significantly higher Chl *b* compared to the WT, even at Day 0. At Day 8, the aabb mutant genotype had the highest Chl *b* content, approximately six times higher than the WT. The chlorophyll *a*/chlorophyll *b* (Chl *a*/Chl *b*) ratio decreased as the number of knockout alleles increased, with the aabb mutant genotype having the lowest value, about 1/3 of the WT (Fig. 3d). Before dark incubation, the photochemical efficiency (Fv/Fm) of aabb mutant genotype was significantly lower than the WT. However, the Fv/Fm value of all mutant genotypes experienced similar reductions as WT during dark treatment as shown in Fig. 3e.

**Fig. 4:**
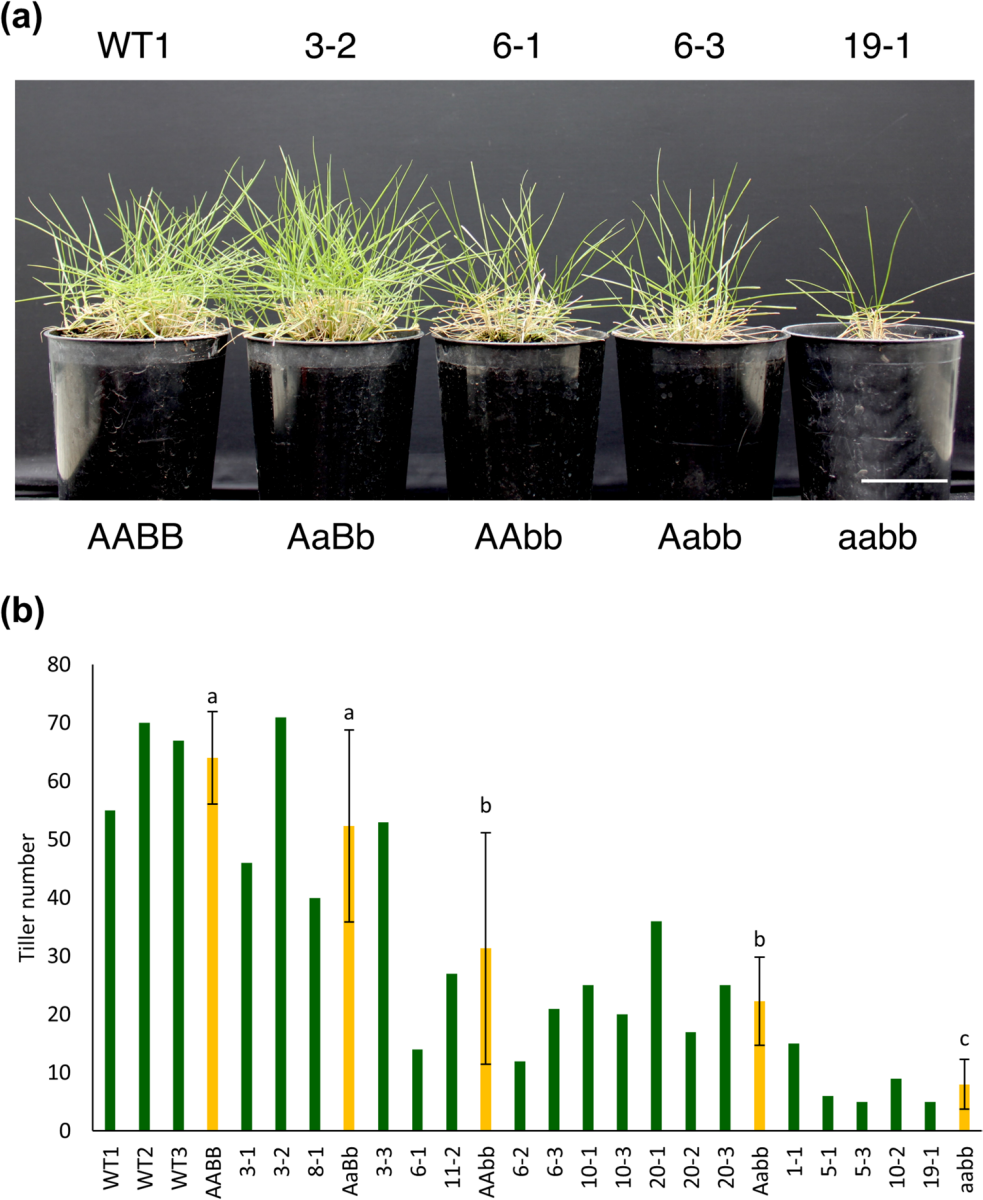
Phenotypic and tiller number comparison between wild type (WT) and *ZmNYC1* mutant lines for each genotype. **a** The representative photos of mutant lines for each genotype compared with wild type (WT). Scale bar: 5 cm. **b** Tiller number. Mean of genotypes are the values of plant lines of the same genotype. Error bars represent the standard deviation (SD). Letters represent significant differences determined by one-way ANOVA with a post hoc LSD test (*P* < 0.05, *n* ≥ 3)

Figure 4a shows the phenotypes of each mutant genotype three months after acclimatization in a GM greenhouse. The mutant genotypes significantly affected plant growth. Plants with the heterozygous AaBb genotype showed similar growth to the WT. The AAbb and Aabb mutant genotypes displayed an intermediate phenotype between the WT and the aabb, with the aabb mutant exhibiting the lowest plant growth among all the observed genotypes. This result was consistent with the tiller number measurements, where the tiller number of the AAbb and Aabb was significantly lower than the WT and AaBb mutant genotype (Fig. 4b). The aabb mutant genotype had significantly lower tiller numbers compared to the WT and the other mutant genotypes. Conversely, plants with the homozygous aabb mutant genotype retained their green color during cultivation, aligning with the previously observed results of chlorophyll content before dark treatment (Fig. 3a).

### Stay-green phenotype of *ZmNYC1* knockout mutant line under simulated winter condition

**Fig. 5:**
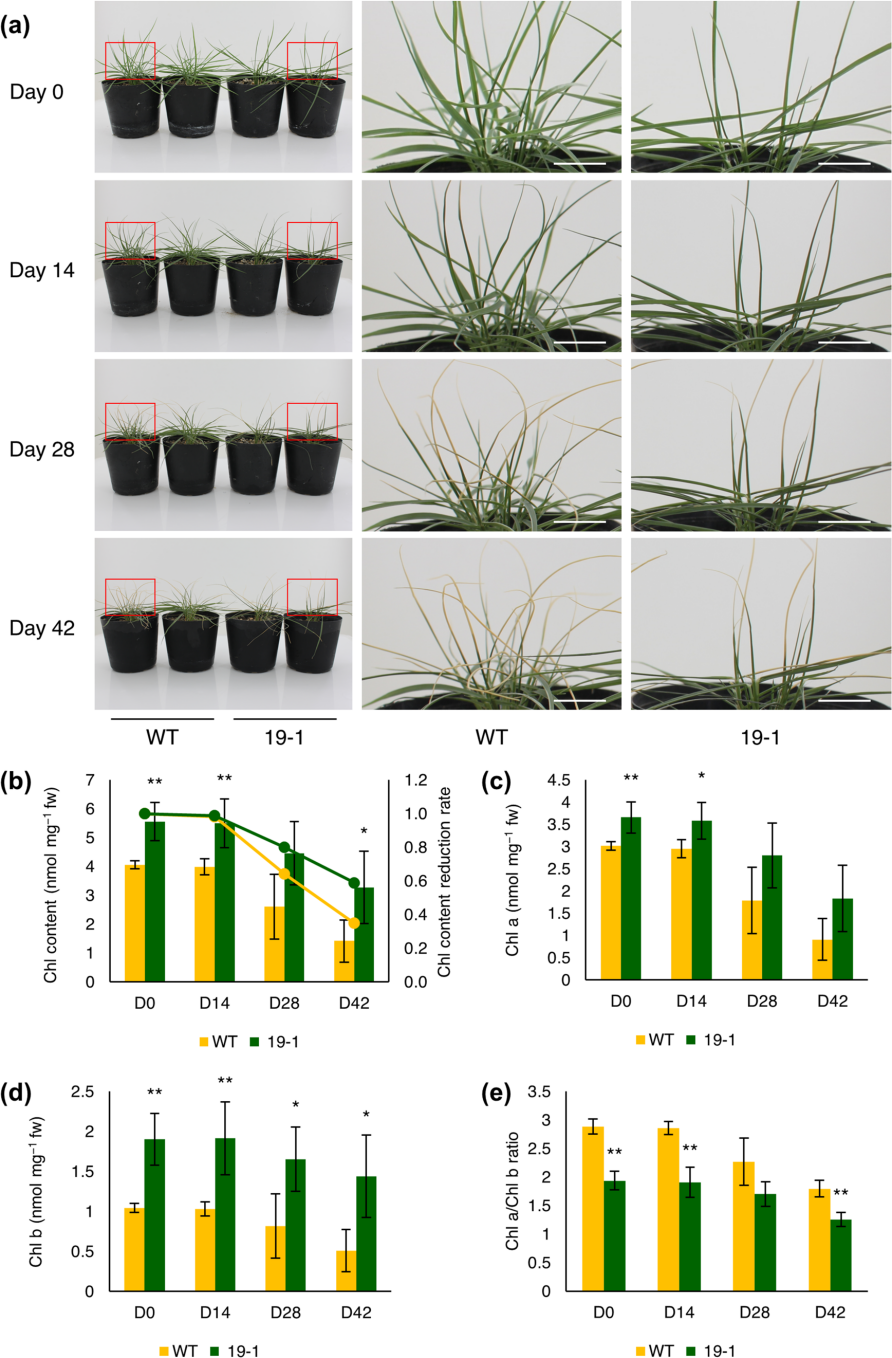
Characteristics of *ZmNYC1* knockout mutant line 19–1 under winter simulation test. **a** Phenotypes of the wild type (WT) and knockout mutant line 19–1 at Day 0, 14, 28, and 42 after winter simulation treatment. Scale bar: 1 cm. **b** Bar chart represents chlorophyll content; line chart represents chlorophyll content reduction rate; WT is indicated by yellow line: line19-1 is indicated by green line, **c** Chlorophyll *a* content, **d** Chlorophyll *b* content, and **e** Chlorophyll *a*/ Chlorophyll* b* ratio for WT and line 19–1 at Day 0, 14, 28, and 42. Error bars represent the standard deviation (SD). Asterisks indicate significant differences on each treatment day determined by Student’s t test (∗ *P* ≤ 0.05 and ∗  ∗ *P* ≤ 0.01, *n* = 4)

To evaluate the evergreen characteristics of the completely knockout line 19–1, we exposed the plants to simulated "cold" winter at 5 °C under a constant short-day photoperiod of 8 h of light and 16 h of darkness. The chlorophyll content of the plants was analyzed every 2 weeks for a duration of up to 42 days. At Day 0, the leaves of line 19–1 were visibly darker green compared to WT (Fig. 5a). After 28 days of treatment, both WT and line 19–1 showed curled leaves and yellowing from the tips of leaves, with the WT leaves exhibiting more yellowing and line 19–1 retaining more greenness. At Day 42, the number of yellowed and curled leaves increased in WT compared to line 19–1. The phenotypes observed in simulated winter condition were linked to chlorophyll contents, with line 19–1 consistently showing higher chlorophyll contents from Day 0 to Day 42 of treatment (Fig. 5b–d). The chlorophyll content reduction trend in line 19–1 was comparable to that of the WT (Fig. 5b). Although there were no significant differences in the chlorophyll reduction rate, line 19–1 retained approximately 60% of chlorophyll content at Day 42, which was almost double that of the WT. Notably, Chl *b* in line 19–1 maintained significantly higher levels throughout the winter simulation treatment, leading to a lower Chl *a*/Chl *b* ratio compared to the WT (Fig. 5e).

## Discussion

Zoysiagrass exhibits high morphological, physiological, and genetic diversity, and its ability for interspecific hybridization has led to the development of numerous varieties with valuable traits (Chandra et al. 2017; Patton et al. 2017). However, due to the absence of the evergreen trait within its diverse genetic resources, conventional breeding methods could not facilitate the development of stay-green varieties. Our research group had previously selected *Z. matrella* ‘Wakaba’ as the superior ecotype from 250 eco-collections for turf quality in Japan and subsequently developed it as a cultivar (Hashiguchi et al. 2006; Tanaka et al. 2016b). We also generated a high-quality draft genome sequence of *Z. japonica*, and draft genome sequences of *Z. matrella* and *Z. pacifica*, with ‘Wakaba’ serving as the representative material for *Z. matrella* (Tanaka et al. 2016a). Additionally, by leveraging the experience of our research group in establishing tissue culture systems for various warm-season grasses (Muguerza et al. 2022), we have established an efficient transformation and tissue culture system using this cultivar (Ng et al. 2023). These collective achievements have laid the foundation for molecular breeding within the *Zoysia* genus, leading to our success in this study by conducting genome editing for the first time in zoysiagrass, targeting the *NYC1* gene responsible for chlorophyll degradation in *Z. matrella* ‘Wakaba’.

Achieving a complete knockout of *ZmNYC1* proved challenging due to the highly heterozygous nature of the allotetraploid *Z. matrella* ‘Wakaba’. The *ZmNYC1* loci consist of two sub-genomes, suggested to be derived from *Z. japonica* (A sub-genome) and *Z. pacifica* (B sub-genome), respectively (Tanaka et al. 2016b, a). We designed a gRNA targeting both A and B sub-genomes and employed the all-in-one vector containing MMCas9 and OsU6-gRNA expression constructs (pZH_OsU6gRNA_MMCas9), given its demonstrated effectiveness in inducing high-frequency mutagenesis in target genes as confirmed in rice and wheat (Mikami et al. 2015; Abe et al. 2019). Despite the relatively low transformation efficiency, we found it to be adequately sufficient for generating genome-edited T0 plants with multiple genotypes in zoysiagrass. Through this approach, we successfully achieved efficient targeted mutagenesis and accomplished the knockout of both sub-genomes of the *ZmNYC1* gene in the T0 generation.

Sequencing analysis of some transformed calli revealed a wide range of mutations, mostly consisting of 1 or 2 bp indels. T0 plants from the same callus lines exhibited a variety of mutations, including those not initially detected in the screened callus line, and several displayed chimeric mutations in both A and B sub-genomes. The phenomenon of enhanced mutation frequency with prolonged culture duration, as seen in rice (Mikami et al. 2015), likely resulted from the continuous subculture of transformed calli prior to plant regeneration, which extended the exposure to CRISPR/Cas9 and contributed to additional mutations. Additionally, regenerated plants may exhibit chimeric mutations if mutations occur after cell division of the transformed embryogenic cell (Zhang et al. 2014). Although cell divisions are lower in regenerated plants than in callus, reducing Cas9/gRNA expression and the likelihood of new mutations, a recent study on apomictic tetraploid bahiagrass demonstrated progressive editing after soil transfer (May et al. 2023), potentially due to the maize ubiquitin promoter driving Cas9 expression, which was also used in this study. This progressive editing complicates the identification of mutation timing in regenerated plants. Therefore, to address this, alternative promoters like tissue-specific or inducible promoters should be considered for spatiotemporal regulation of Cas9 expression in future studies (Rahman et al. 2022). Another alternative approach is the introduction of Cas9-gRNA ribonucleoproteins which can radically solve the problem of using DNA vectors and reduce the risk of off-target, and is expected to be developed in the future (Zhang et al. 2021).

In this study, the genome-edited *ZmNYC1* in *Z. matrella* displayed stay-green phenotypes following dark-induced senescence. Moreover, the T0 plants with the aabb mutant genotype showed a visually darker green color than the WT even before treatments showcasing a superior stay-green trait. These results are consistent with previous studies that demonstrated mutants of the *NYC1* gene in Arabidopsis (Jibran et al. 2015) and rice (Kusaba et al. 2007) retained greenness in leaves after induced senescence by dark treatment. The *ZmNYC1* T0 lines also showed higher levels of total chlorophyll, Chl *a*, and particularly Chl *b*, after dark treatment, leading to a lower Chl *a*/Chl *b* ratio, consistent with the rice *NYC1* mutant (Kusaba et al. 2007). These results suggest that the targeted mutations effectively disrupted the functionality of the *ZmNYC1* gene. The Fv/Fm of *ZmNYC1* mutant lines declined similarly to the WT during dark treatment. Higher chlorophyll content in *NYC1* mutant does not necessarily imply a higher photosynthesis ability, as *NYC1* is involved in chlorophyll degradation, not leaf longevity (Kusaba et al. 2007). Hence, the *ZmNYC1* mutant is classified as a cosmetic stay-green mutant, retaining green leaf color while exhibiting a loss in photosynthetic capacity (Thomas and Howarth 2000).

In addition to exhibiting a stay-green phenotype, the *ZmNYC1* mutation also inhibited plant growth in homozygous mutant genotypes, leading to reduced tiller numbers (Fig. 4). This contrasts with findings in other plant species with *NYC1* mutants, as no compromised yield or fitness was observed except for reduced seed germination in the nyc1/nol *Arabidopsis* mutant (Nakajima et al. 2012; Jibran et al. 2015). This result might be associated with the complex regulation of the chlorophyll cycle, responsible for the interconversion between Chl *a* and Chl *b*, which is crucial for maintaining the balance of the Chl *a*/Chl *b* ratio under different physiological conditions (Ito et al. 1996). In this study, the Chl *a*/Chl *b* ratio for WT was generally above 2.5 before treatment. However, a lower Chl *a*/Chl *b* ratio became more pronounced with an increased number of mutated alleles, correlating with the observed reduction in tiller growth. This indicates the importance of the chlorophyll cycle in regulating the Chl *a*/Chl *b* ratio, an important determinant of light absorption efficiency of photosynthesis and its overall impact on plant growth. Additionally, mutants with suppressed Chl *b* degradation may hinder the degradation of LHCII, which is the most abundant membrane protein, potentially disrupting the efficient allocation of nitrogen and carbon to sink organs (Kusaba et al. 2007; Horie et al. 2009). Moreover, accumulated LHCII in these mutant genotypes may result in increased light capture but cannot fully utilize the absorbed light energy for photosynthesis because of the reduced amount of the PSII core complexes (Kusaba et al. 2007). As a result, the Fv/Fm ratio, which serves as an indicator of maximum photochemical efficiency of PSII, was significantly lower in the aabb mutant genotype compared to the WT, indicating reduced photosynthetic efficiency. Furthermore, leaves of this mutant genotype may have been causing cell death as a result of reactive oxygen species (ROS) generated by excess chlorophyll (Hu et al. 2021). Among the four mutant genotypes observed, the complete homozygous mutant exhibited the lowest growth, whereas the AAbb and Aabb mutant genotypes with the homozygous sub-genome B showed moderate greenness and growth between the complete knockout mutant and the WT. Consequently, a moderately *ZmNYC1*-edited mutant that retains a stay-green phenotype without adversely affecting growth is likely a more suitable candidate for practical applications. The removal of transgene and screening of null-segregant in these candidates will be necessary to delimit Cas9 expression and prevent additional mutations in these mutant genotypes for future breeding.

Under simulated winter conditions, the aabb mutant genotype exhibited better preservation of greenness, with significantly higher chlorophyll content, particularly Chl *b*, compared to the WT. This highlights the potential of the *ZmNYC1* knockout approach to mitigate the reduction in greenness during winter dormancy in zoysiagrass. Nevertheless, the aabb mutant genotype displayed gradual chlorophyll decline, indicating the potential upregulation of other genes involved in photosynthesis, carbon assimilation, ROS production, and hormone synthesis under cold conditions and reduced photoperiods (Teng et al. 2016, 2021). This observation aligns with a recent RNA-seq analysis of zoysiagrass under cold stress, which revealed the down-regulation of genes associated with chlorophyll biosynthesis and chlorophyll *a*/*b* binding, alongside the upregulation of six genes related to chlorophyll catabolism (Wei et al. 2015; Long et al. 2020). The combination of low temperature with high light has the potential to induce chronic photoinhibition of PSII in warm-season plants (Allen and Ort 2001). This inhibition was observed in both WT and line 19–1 mutant under simulated winter conditions, as the Fv/Fm values were zero after one week. While the *ZmNYC1* mutant shows promise in maintaining a green color during winter, we consider it is also essential to identify the key genes directly involved in winter dormancy. This first established genome editing technology for *Z. matrella* can be effectively applied to the functional analysis of novel genes and the development of new varieties of zoysiagrass in the future.

In conclusion, we successfully applied the CRISPR/Cas9 system in the highly heterozygous allotetraploid *Z. matrella* ‘Wakaba’ using our previously established stable *Agrobacterium* transformation system (Ng et al. 2023). Specifically, we targeted the *ZmNYC1* loci and obtained a total knockout of all four *ZmNYC1* alleles in the T0 generation. These mutants exhibited a longer stay-green phenotype than the WT under dark-induced and winter simulation treatments. We believe that the mutants developed in this study hold the promise of being utilized as novel breeding materials capable of maintaining greenness for extended periods during winter. In the future, off-target screening and the removal of transgenes by self-pollination will enable breeding development with a view to practical application.

### Supplementary Information

Below is the link to the electronic supplementary material.

Supplementary file1 (PDF 939 KB)

## Data Availability

The original contributions presented in the study are included in the article/Supplementary Material, further inquiries can be directed to the corresponding author.
