# Supplementary material for: CRISPR/Cas9-mediated knockout of NYC1 gene enhances chlorophyll retention and reduces tillering in Zoysia matrella (L.) Merrill
Source: Plant Cell Rep. 2024 Feb 2;43(2):50. doi: 10.1007/s00299-023-03130-6 (PMC10837251; doi:10.1007/s00299-023-03130-6)
Supplement: Supplementary file 1 — Supplementary file1 (PDF 939 KB) [file 299_2023_3130_MOESM1_ESM.pdf]

Plant Cell Reports

Supplementary Material

CRISPR/Cas9-mediated knockout of *NYC1* gene enhances chlorophyll retention and reduces tillering in *Zoysia matrella* (L.) Merrill

Hwan May Ng, Takahiro Gondo *, Hidenori Tanaka, Ryo Akashi

Correspondence

Takahiro Gondo, Frontier Science Research Center, University of Miyazaki, 1-1 Gakuenkibanadai-Nishi, Miyazaki 889-2192, Japan.

E-mail: gondo@cc.miyazaki-u.ac.jp

Phone number: +81-985-58-7168

Fax number: +81-985-58-7761

```
ZmNYC1_A   1:AGGTTGTTTCAACGTGTGGCCACCTCGTTTCTTCCTTATTTATTCACACGTACTCCGTAG 60
ZmNYC1_B   1:AGGTTGTTTCAACGTGTGGCCACCTCGTTTCTTCCTTATTTATTCACACGTACTCCGTAG 60
             ************************************************************

ZmNYC1_A  61:TAGGAATTCACTGGAGTGGACGAGGCAAAAGTGCCCGGCACACGCCTCTCCTCTCCTAAA 120
ZmNYC1_B  61:TAC-------------TGGACGAGGCAAAAGTGCCCGGCACACGCCTTTCCTCTCCTAAA 107
             **              ******************************* ************

ZmNYC1_A 121:ATTCCGGGAGAAAAAGAAAAAAAAAA------GACGACGACGACAGAAGCACACGCAACT 174
ZmNYC1_B 108:ATTCCGGGAGAAAAAGAAAAAAAAAAAAAAAAGACGACGACGAGAGAAGCACACGCAACT 167
             *************************       *********** ****************

ZmNYC1_A 175:ACTCCCTCGTCCCGACCTTATCCGCGCCATGGCCGCCGCCGCCGTCGCGCACCTCTCCGT 234
ZmNYC1_B 168:ACTCCCTCGTCCCGACCTTATCCGCGCCATGGCCGCCGCCG---TCGCGCACCTCTCCGT 224
             *****************************************   ****************

ZmNYC1_A 235:CCACGGCCGCCCCCGCCGCTCGCCGGACCTGCTCTCGACCTCCTCGTGCCGGCCACGGCC 294
ZmNYC1_B 225:CCACGGCCGCCCCCGCCGCTCGCCGGACCTGCTCTCGACCTCCTCGTGCCGGCCACGGCC 284
             ************************************************************

ZmNYC1_A 295:CTCTCTCCTCCGCTGCCGCGCGTTCAAGCAGGAGCCCCCCGTCGACCGCGACCCGTCGCC 354
ZmNYC1_B 285:CTCTCTCCTCCGCTGCCGCGCGTTCAAGCAGGAGCCCCCCGTCGACCGCGACCCGTCGCC 344
             ************************************************************

ZmNYC1_A 355:GACGCCGCCCGAGCCGCGGAAGCACAGGAAGGGCCCGCTCTACAAGCTCAAGGCCGCGAT 414
ZmNYC1_B 345:GACGCCGCCCGAGCCGCGGAAGCACAGGAAGGGCCCGCTCTACAAGCTCAAGGCCGCGAT 404
             ************************************************************

ZmNYC1_A 415:TCAGGGGCTAGCGGGCTCACGGACGGCGGCCGCGGAGGTCTATGGCGGGGAGTA       468
ZmNYC1_B 405:TCAGGGGCTAGCGGGCTCACGGACGGCGGCCGCGGAGGTCTATGGCGGGGAGTA       458
             ******************************************************
```

Fig. S1 Related to Fig. 1c

Sequences of the amplified region of heterozygous *ZmNYC1* target loci using the common primer for A and B sub-genomes (*ZmNYC1*_AB), related to Fig. 1c. The guide RNA (gRNA) is indicated by black line. The protospacer-adjacent motif sequence (PAM) is shown in blue, and the *Hha*I restriction site is shown in red. Asterisk indicates an identical nucleotide and a dash indicates a gap. The amplified sequence length of *ZmNYC1*_A (A sub-genome) is 468 bp while *ZmNYC1*_B (B sub-genome) is 458 bp. The *Hha*I-digested fragment sizes of A sub-genome are 23, 91, 154, and 200 bp and B sub-genome are 20, 91, 154, and 193 bp. When mutation is inserted 2 to 4 bp upstream of the PAM sequence, a detectable band at around 111/114-bp can be seen as shown in Fig. 1c. The homoduplex bands (~200-bp) seen in the CAPS analysis, as shown in Fig. 1c, are composed of the 200 and 193 bp fragments. Heteroduplex bands above it (~242-bp and ~300-bp) form due to a 22 bp mismatch between the 200 and 193 bp fragments.

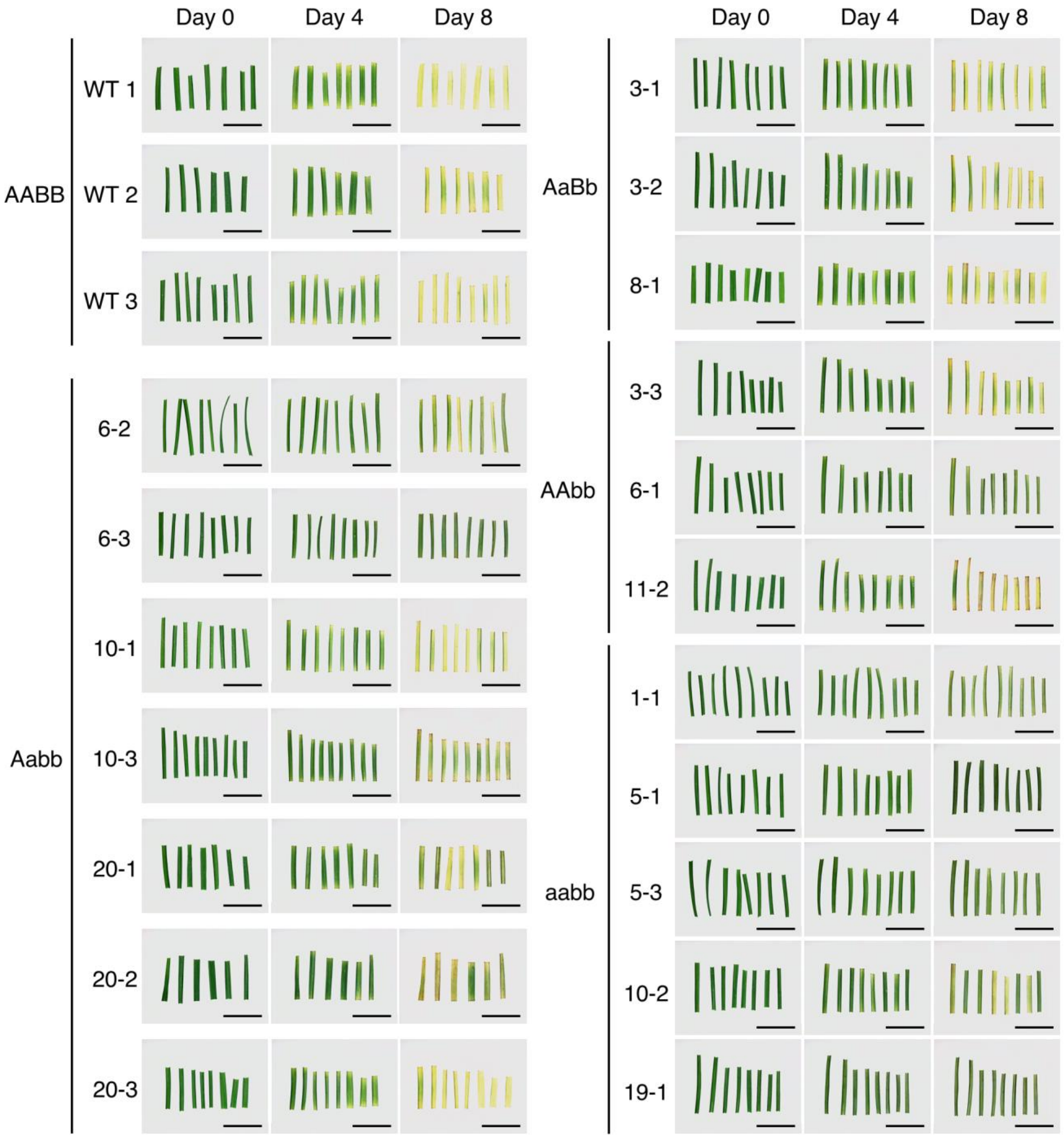

Fig. S2 Phenotypic comparison of detached leaves between wild type (WT) and *ZmNYC1* mutant lines for each genotype during dark-induced senescence at Day 0, 4 and 8. Plants are identified by Line-Plant number, and they are categorized by their respective mutant genotypes. Scale bar: 1 cm.

Table S1 PCR primers used in this study (*ZmNYC1*).

Name	Direction	Sequence	Fragment size (bp)	Application
*ZmNYC1*_A	F	TGGAGTGGACGAGGCAAAAG	397	Amplification of *ZmNYC1* target site in A sub-genome
	R	TACTCCCCGCCATAGACCTC		
*ZmNYC1*_B	F	CGTAGTACTGGACGAGGCAA	403	Amplification of *ZmNYC1* target site in B sub-genome
	R	TACTCCCCGCCATAGACCTC		
*ZmNYC1*_AB	F	AGGTTGTTTCAACGTGTGGC	468	Amplification of *ZmNYC1* target site in A and B sub-genomes
	R	TACTCCCCGCCATAGACCTC		

Table S2 Data generated by the ICE software based on Sanger sequencing of transformed calli, related to Table 2.

<table>
<tr><th>Callus Line</th><th>Allele</th><th>KO-score</th><th>Indel (bp) [†]</th><th>Indel ratio</th><th>Sequence [‡]</th></tr>
<tr><td rowspan="6">1</td><td rowspan="3">A</td><td rowspan="3">21</td><td>-2</td><td>5%</td><td>GGGCGGCCGTGGACGGAGAGGTGCG|--ACGGCGGCGGCCATGGCGCGGATAAGGTCGGGACGAGGGAGTAGT</td></tr>
<tr><td>-1</td><td>16%</td><td>GGGCGGCCGTGGACGGAGAGGTGC-|CGACGGCGGCGGCCATGGCGCGGATAAGGTCGGGACGAGGGAGTAGT</td></tr>
<tr><td>0</td><td>73%</td><td>GGGCGGCCGTGGACGGAGAGGTGCG|CGACGGCGGCGGCCATGGCGCGGATAAGGTCGGGACGAGGGAGTAGT</td></tr>
<tr><td rowspan="3">B</td><td rowspan="3">83</td><td>-1</td><td>42%</td><td>GGGCGGCCGTGGACGGAGAGGTGC-|CGACGGCGGCCATGGCGCGGATAAGGTCGGGACGAGGGAGTAGTTGC</td></tr>
<tr><td>0</td><td>13%</td><td>GGGCGGCCGTGGACGGAGAGGTGCG|CGACGGCGGCCATGGCGCGGATAAGGTCGGGACGAGGGAGTAGTTGC</td></tr>
<tr><td>+1</td><td>41%</td><td>GGGCGGCCGTGGACGGAGAGGTGCG|nCGACGGCGGCCATGGCGCGGATAAGGTCGGGACGAGGGAGTAGTTG</td></tr>
<tr><td rowspan="2">2</td><td>A</td><td>0</td><td>0</td><td>100%</td><td>GGGCGGCCGTGGACGGAGAGGTGCG|CGACGGCGGCGGCCATGGCGCGGATAAGGTCGGGACGAGGGAGTAGT</td></tr>
<tr><td>B</td><td>0</td><td>0</td><td>95%</td><td>GGGCGGCCGTGGACGGAGAGGTGCG|CGACGGCGGCCATGGCGCGGATAAGGTCGGGACGAGGGAGTAGTTGC</td></tr>
<tr><td rowspan="6">3</td><td rowspan="3">A</td><td rowspan="3">41</td><td>-2</td><td>35%</td><td>GGGCGGCCGTGGACGGAGAGGTGCG|--ACGGCGGCGGCCATGGCGCGGATAAGGTCGGGACGAGGGAGTAGT</td></tr>
<tr><td>-1</td><td>6%</td><td>GGGCGGCCGTGGACGGAGAGGTGC-|CGACGGCGGCGGCCATGGCGCGGATAAGGTCGGGACGAGGGAGTAGT</td></tr>
<tr><td>0</td><td>55%</td><td>GGGCGGCCGTGGACGGAGAGGTGCG|CGACGGCGGCGGCCATGGCGCGGATAAGGTCGGGACGAGGGAGTAGT</td></tr>
<tr><td rowspan="3">B</td><td rowspan="3">49</td><td>-1</td><td>20%</td><td>GGGCGGCCGTGGACGGAGAGGTGC-|CGACGGCGGCCATGGCGCGGATAAGGTCGGGACGAGGGAGTAGTTGC</td></tr>
<tr><td>0</td><td>46%</td><td>GGGCGGCCGTGGACGGAGAGGTGCG|CGACGGCGGCCATGGCGCGGATAAGGTCGGGACGAGGGAGTAGTTGC</td></tr>
<tr><td>+1</td><td>29%</td><td>GGGCGGCCGTGGACGGAGAGGTGCG|nCGACGGCGGCCATGGCGCGGATAAGGTCGGGACGAGGGAGTAGTTG</td></tr>
<tr><td rowspan="2">4</td><td>A</td><td>0</td><td>0</td><td>100%</td><td>GGGCGGCCGTGGACGGAGAGGTGCG|CGACGGCGGCGGCCATGGCGCGGATAAGGTCGGGACGAGGGAGTAGT</td></tr>
<tr><td>B</td><td>0</td><td>0</td><td>100%</td><td>GGGCGGCCGTGGACGGAGAGGTGCG|CGACGGCGGCCATGGCGCGGATAAGGTCGGGACGAGGGAGTAGTTGC</td></tr>
<tr><td rowspan="7">5</td><td rowspan="3">A</td><td rowspan="3">46</td><td>-7</td><td>6%</td><td>GGGCGGCCGTGGACGGAGAG-----|--ACGGCGGCGGCCATGGCGCGGATAAGGTCGGGACGAGGGAGTAGT</td></tr>
<tr><td>0</td><td>48%</td><td>GGGCGGCCGTGGACGGAGAGGTGCG|CGACGGCGGCGGCCATGGCGCGGATAAGGTCGGGACGAGGGAGTAGT</td></tr>
<tr><td>+1</td><td>40%</td><td>GGGCGGCCGTGGACGGAGAGGTGCG|nCGACGGCGGCGGCCATGGCGCGGATAAGGTCGGGACGAGGGAGTAG</td></tr>
<tr><td rowspan="4">B</td><td rowspan="4">58</td><td>-2</td><td>30%</td><td>GGGCGGCCGTGGACGGAGAGGTGCG|--ACGGCGGCCATGGCGCGGATAAGGTCGGGACGAGGGAGTAGTTGC</td></tr>
<tr><td>-1</td><td>10%</td><td>GGGCGGCCGTGGACGGAGAGGTGC-|CGACGGCGGCCATGGCGCGGATAAGGTCGGGACGAGGGAGTAGTTGC</td></tr>
<tr><td>0</td><td>38%</td><td>GGGCGGCCGTGGACGGAGAGGTGCG|CGACGGCGGCCATGGCGCGGATAAGGTCGGGACGAGGGAGTAGTTGC</td></tr>
<tr><td>+1</td><td>18%</td><td>GGGCGGCCGTGGACGGAGAGGTGCG|nCGACGGCGGCCATGGCGCGGATAAGGTCGGGACGAGGGAGTAGTTG</td></tr>
<tr><td rowspan="3">6</td><td rowspan="3">A</td><td rowspan="3">59</td><td>-1</td><td>40%</td><td>GGGCGGCCGTGGACGGAGAGGTGC-|CGACGGCGGCGGCCATGGCGCGGATAAGGTCGGGACGAGGGAGTAGT</td></tr>
<tr><td>0</td><td>33%</td><td>GGGCGGCCGTGGACGGAGAGGTGCG|CGACGGCGGCGGCCATGGCGCGGATAAGGTCGGGACGAGGGAGTAGT</td></tr>
<tr><td>+1</td><td>19%</td><td>GGGCGGCCGTGGACGGAGAGGTGCG|nCGACGGCGGCGGCCATGGCGCGGATAAGGTCGGGACGAGGGAGTAG</td></tr>
</table>

Table S2 Data generated by the ICE software based on Sanger sequencing of transformed calli, related to Table 2. (Continued)

<table>
<thead>
<tr><th>Callus Line</th><th>Allele</th><th>KO-score</th><th>Indel (bp) †</th><th>Indel ratio</th><th>Sequence ‡</th></tr>
</thead>
<tbody>
<tr><td rowspan="3">6</td><td rowspan="3">B</td><td rowspan="3">90</td><td>-1</td><td>25%</td><td>GGGCGGCCGTGGACGGAGAGGTGC-|CGACGGCGGCGGCCATGGCGCGGATAAGGTCGGGACGAGGGAGTAGTTGC</td></tr>
<tr><td>0</td><td>7%</td><td>GGGCGGCCGTGGACGGAGAGGTGCG|CGACGGCGGCGGCCATGGCGCGGATAAGGTCGGGACGAGGGAGTAGTTGC</td></tr>
<tr><td>+1</td><td>65%</td><td>GGGCGGCCGTGGACGGAGAGGTGCG|nCGACGGCGGCGGCCATGGCGCGGATAAGGTCGGGACGAGGGAGTAGTTG</td></tr>
<tr><td rowspan="3">7</td><td>A</td><td>0</td><td>0</td><td>99%</td><td>GGGCGGCCGTGGACGGAGAGGTGCG|CGACGGCGGCGGCGGCCATGGCGCGGATAAGGTCGGGACGAGGGAGTAGT</td></tr>
<tr><td rowspan="2">B</td><td rowspan="2">27</td><td>0</td><td>71%</td><td>GGGCGGCCGTGGACGGAGAGGTGCG|CGACGGCGGCGGCCATGGCGCGGATAAGGTCGGGACGAGGGAGTAGTTGC</td></tr>
<tr><td>+1</td><td>27%</td><td>GGGCGGCCGTGGACGGAGAGGTGCG|nCGACGGCGGCGGCCATGGCGCGGATAAGGTCGGGACGAGGGAGTAGTTG</td></tr>
<tr><td rowspan="5">8</td><td rowspan="2">A</td><td rowspan="2">30</td><td>-1</td><td>30%</td><td>GGGCGGCCGTGGACGGAGAGGTGC-|CGACGGCGGCGGCGGCCATGGCGCGGATAAGGTCGGGACGAGGGAGTAGT</td></tr>
<tr><td>0</td><td>68%</td><td>GGGCGGCCGTGGACGGAGAGGTGCG|CGACGGCGGCGGCGGCCATGGCGCGGATAAGGTCGGGACGAGGGAGTAGT</td></tr>
<tr><td rowspan="3">B</td><td rowspan="3">27</td><td>-1</td><td>5%</td><td>GGGCGGCCGTGGACGGAGAGGTGC-|CGACGGCGGCGGCCATGGCGCGGATAAGGTCGGGACGAGGGAGTAGTTGC</td></tr>
<tr><td>0</td><td>71%</td><td>GGGCGGCCGTGGACGGAGAGGTGCG|CGACGGCGGCGGCCATGGCGCGGATAAGGTCGGGACGAGGGAGTAGTTGC</td></tr>
<tr><td>+1</td><td>22%</td><td>GGGCGGCCGTGGACGGAGAGGTGCG|nCGACGGCGGCGGCCATGGCGCGGATAAGGTCGGGACGAGGGAGTAGTTG</td></tr>
<tr><td rowspan="4">9</td><td rowspan="2">A</td><td rowspan="2">5</td><td>0</td><td>92%</td><td>GGGCGGCCGTGGACGGAGAGGTGCG|CGACGGCGGCGGCGGCCATGGCGCGGATAAGGTCGGGACGAGGGAGTAGT</td></tr>
<tr><td>+1</td><td>5%</td><td>GGGCGGCCGTGGACGGAGAGGTGCG|nCGACGGCGGCGGCGGCCATGGCGCGGATAAGGTCGGGACGAGGGAGTAG</td></tr>
<tr><td rowspan="2">B</td><td rowspan="2">6</td><td>0</td><td>92%</td><td>GGGCGGCCGTGGACGGAGAGGTGCG|CGACGGCGGCGGCCATGGCGCGGATAAGGTCGGGACGAGGGAGTAGTTGC</td></tr>
<tr><td>+1</td><td>6%</td><td>GGGCGGCCGTGGACGGAGAGGTGCG|nCGACGGCGGCGGCCATGGCGCGGATAAGGTCGGGACGAGGGAGTAGTTG</td></tr>
<tr><td rowspan="4">10</td><td>A</td><td>96</td><td>-2</td><td>96%</td><td>GGGCGGCCGTGGACGGAGAGGTGCG|--ACGGCGGCGGCGGCCATGGCGCGGATAAGGTCGGGACGAGGGAGTAGT</td></tr>
<tr><td rowspan="3">B</td><td rowspan="3">23</td><td>-1</td><td>13%</td><td>GGGCGGCCGTGGACGGAGAGGTGC-|CGACGGCGGCGGCCATGGCGCGGATAAGGTCGGGACGAGGGAGTAGTTGC</td></tr>
<tr><td>0</td><td>74%</td><td>GGGCGGCCGTGGACGGAGAGGTGCG|CGACGGCGGCGGCCATGGCGCGGATAAGGTCGGGACGAGGGAGTAGTTGC</td></tr>
<tr><td>+1</td><td>10%</td><td>GGGCGGCCGTGGACGGAGAGGTGCG|nCGACGGCGGCGGCCATGGCGCGGATAAGGTCGGGACGAGGGAGTAGTTG</td></tr>
<tr><td rowspan="2">11</td><td>A</td><td>0</td><td>0</td><td>95%</td><td>GGGCGGCCGTGGACGGAGAGGTGCG|CGACGGCGGCGGCGGCCATGGCGCGGATAAGGTCGGGACGAGGGAGTAGT</td></tr>
<tr><td>B</td><td>98</td><td>+1</td><td>98%</td><td>GGGCGGCCGTGGACGGAGAGGTGCG|nCGACGGCGGCGGCCATGGCGCGGATAAGGTCGGGACGAGGGAGTAGTTG</td></tr>
<tr><td rowspan="3">12</td><td>A</td><td>0</td><td>0</td><td>100%</td><td>GGGCGGCCGTGGACGGAGAGGTGCG|CGACGGCGGCGGCGGCCATGGCGCGGATAAGGTCGGGACGAGGGAGTAGT</td></tr>
<tr><td rowspan="2">B</td><td rowspan="2">9</td><td>0</td><td>88%</td><td>GGGCGGCCGTGGACGGAGAGGTGCG|CGACGGCGGCGGCCATGGCGCGGATAAGGTCGGGACGAGGGAGTAGTTGC</td></tr>
<tr><td>+1</td><td>9%</td><td>GGGCGGCCGTGGACGGAGAGGTGCG|nCGACGGCGGCGGCCATGGCGCGGATAAGGTCGGGACGAGGGAGTAGTTG</td></tr>
<tr><td rowspan="2">13</td><td>A</td><td>0</td><td>0</td><td>100%</td><td>GGGCGGCCGTGGACGGAGAGGTGCG|CGACGGCGGCGGCGGCCATGGCGCGGATAAGGTCGGGACGAGGGAGTAGT</td></tr>
<tr><td>B</td><td>0</td><td>0</td><td>100%</td><td>GGGCGGCCGTGGACGGAGAGGTGCG|CGACGGCGGCGGCCATGGCGCGGATAAGGTCGGGACGAGGGAGTAGTTGC</td></tr>
</tbody>
</table>

Table S2 Data generated by the ICE software based on Sanger sequencing of transformed calli, related to Table 2. (Continued)

Callus Line	Allele	KO-score	Indel (bp) †	Indel ratio	Sequence ‡
14	A	0	0	92%	GGGCGGCCGTGGACGGAGAGGTGCG|CGACGGCGGCGGCGGCCATGGCGCGGATAAGGTCGGGACGAGGGAGTAGT
	B	0	-6	89%	GGGCGGCCGTGGACGGAGAGGTGCG|------CGGCGGCCATGGCGCGGATAAGGTCGGGACGAGGGAGTAGTTGC
			0	10%	GGGCGGCCGTGGACGGAGAGGTGCG|CGACGGCGGCGGCCATGGCGCGGATAAGGTCGGGACGAGGGAGTAGTTGC
15	A	0	0	100%	GGGCGGCCGTGGACGGAGAGGTGCG|CGACGGCGGCGGCGGCCATGGCGCGGATAAGGTCGGGACGAGGGAGTAGT
	B	0	0	94%	GGGCGGCCGTGGACGGAGAGGTGCG|CGACGGCGGCGGCCATGGCGCGGATAAGGTCGGGACGAGGGAGTAGTTGC
16	A	0	-3	11%	GGGCGGCCGTGGACGGAGAGGTGC-|--ACGGCGGCGGCGGCCATGGCGCGGATAAGGTCGGGACGAGGGAGTAGT
			-3	56%	GGGCGGCCGTGGACGGAGAGGT---|CGACGGCGGCGGCGGCCATGGCGCGGATAAGGTCGGGACGAGGGAGTAGT
			0	21%	GGGCGGCCGTGGACGGAGAGGTGCG|CGACGGCGGCGGCGGCCATGGCGCGGATAAGGTCGGGACGAGGGAGTAGT
	B	98	+1	98%	GGGCGGCCGTGGACGGAGAGGTGCG|nCGACGGCGGCGGCCATGGCGCGGATAAGGTCGGGACGAGGGAGTAGTTG
17	A	0	0	99%	GGGCGGCCGTGGACGGAGAGGTGCG|CGACGGCGGCGGCGGCCATGGCGCGGATAAGGTCGGGACGAGGGAGTAGT
	B	0	0	94%	GGGCGGCCGTGGACGGAGAGGTGCG|CGACGGCGGCGGCCATGGCGCGGATAAGGTCGGGACGAGGGAGTAGTTGC
18	A	0	0	100%	GGGCGGCCGTGGACGGAGAGGTGCG|CGACGGCGGCGGCGGCCATGGCGCGGATAAGGTCGGGACGAGGGAGTAGT
	B	0	0	99%	GGGCGGCCGTGGACGGAGAGGTGCG|CGACGGCGGCGGCCATGGCGCGGATAAGGTCGGGACGAGGGAGTAGTTGC
19	A	98	-1	98%	GGGCGGCCGTGGACGGAGAGGTGC-|CGACGGCGGCGGCGGCCATGGCGCGGATAAGGTCGGGACGAGGGAGTAGT
	B	97	-1	30%	GGGCGGCCGTGGACGGAGAGGTGC-|CGACGGCGGCGGCCATGGCGCGGATAAGGTCGGGACGAGGGAGTAGTTGC
			+1	67%	GGGCGGCCGTGGACGGAGAGGTGCG|nCGACGGCGGCGGCCATGGCGCGGATAAGGTCGGGACGAGGGAGTAGTTG
20	A	5	-2	5%	GGGCGGCCGTGGACGGAGAGGTGCG|--ACGGCGGCGGCGGCCATGGCGCGGATAAGGTCGGGACGAGGGAGTAGT
			0	94%	GGGCGGCCGTGGACGGAGAGGTGCG|CGACGGCGGCGGCGGCCATGGCGCGGATAAGGTCGGGACGAGGGAGTAGT
	B	89	0	8%	GGGCGGCCGTGGACGGAGAGGTGCG|CGACGGCGGCGGCCATGGCGCGGATAAGGTCGGGACGAGGGAGTAGTTGC
			+1	89%	GGGCGGCCGTGGACGGAGAGGTGCG|nCGACGGCGGCGGCCATGGCGCGGATAAGGTCGGGACGAGGGAGTAGTTG
21	A	0	0	100%	GGGCGGCCGTGGACGGAGAGGTGCG|CGACGGCGGCGGCGGCCATGGCGCGGATAAGGTCGGGACGAGGGAGTAGT
	B	0	0	99%	GGGCGGCCGTGGACGGAGAGGTGCG|CGACGGCGGCGGCCATGGCGCGGATAAGGTCGGGACGAGGGAGTAGTTGC
22	A	0	0	100%	GGGCGGCCGTGGACGGAGAGGTGCG|CGACGGCGGCGGCGGCCATGGCGCGGATAAGGTCGGGACGAGGGAGTAGT
	B	0	0	100%	GGGCGGCCGTGGACGGAGAGGTGCG|CGACGGCGGCGGCCATGGCGCGGATAAGGTCGGGACGAGGGAGTAGTTGC

† Indels indicate -; deletion, +; insertion and the number of nucleotides.

‡ Dashes represent deletions and N represents insertion at the guide RNA region. Vertical line represents the expected cut site.

Table S3 Data generated by the ICE software based on Sanger sequencing of regenerated plants.

<table>
<tr><th>Line</th><th>Plant</th><th>Allele</th><th>KO-score</th><th>Indel (bp) †</th><th>Indel ratio</th><th>Sequence ‡</th></tr>
<tr><td rowspan="12">1</td><td rowspan="3">1</td><td rowspan="2">A</td><td rowspan="2">91</td><td>-1</td><td>78%</td><td>GGGCGGCCGTGGACGGAGAGGTGC-|CGACGGCGGCGGCGGCCATGGCGCGGATAAGGTCGGGACGAGGGAGTAGT</td></tr>
<tr><td>+1</td><td>13%</td><td>GGGCGGCCGTGGACGGAGAGGTGCG|nCGACGGCGGCGGCGGCCATGGCGCGGATAAGGTCGGGACGAGGGAGTAG</td></tr>
<tr><td>B</td><td>99</td><td>-1</td><td>99%</td><td>GGGCGGCCGTGGACGGAGAGGTGC-|CGACGGCGGCGGCCATGGCGCGGATAAGGTCGGGACGAGGGAGTAGTTGC</td></tr>
<tr><td rowspan="5">2</td><td rowspan="3">A</td><td rowspan="3">91</td><td>-4</td><td>22%</td><td>GGGCGGCCGTGGACGGAGAGGTGC-|---CGGCGGCGGCGGCCATGGCGCGGATAAGGTCGGGACGAGGGAGTAGT</td></tr>
<tr><td>-4</td><td>24%</td><td>GGGCGGCCGTGGACGGAGAGG----|CGACGGCGGCGGCGGCCATGGCGCGGATAAGGTCGGGACGAGGGAGTAGT</td></tr>
<tr><td>-1</td><td>45%</td><td>GGGCGGCCGTGGACGGAGAGGTGC-|CGACGGCGGCGGCGGCCATGGCGCGGATAAGGTCGGGACGAGGGAGTAGT</td></tr>
<tr><td rowspan="2">B</td><td rowspan="2">33</td><td>0</td><td>61%</td><td>GGGCGGCCGTGGACGGAGAGGTGCG|CGACGGCGGCGGCCATGGCGCGGATAAGGTCGGGACGAGGGAGTAGTTGC</td></tr>
<tr><td>+1</td><td>33%</td><td>GGGCGGCCGTGGACGGAGAGGTGCG|nCGACGGCGGCGGCCATGGCGCGGATAAGGTCGGGACGAGGGAGTAGTTG</td></tr>
<tr><td rowspan="4">3</td><td rowspan="2">A</td><td rowspan="2">99</td><td>-2</td><td>14%</td><td>GGGCGGCCGTGGACGGAGAGGTGCG|--ACGGCGGCGGCGGCCATGGCGCGGATAAGGTCGGGACGAGGGAGTAGT</td></tr>
<tr><td>-1</td><td>85%</td><td>GGGCGGCCGTGGACGGAGAGGTGC-|CGACGGCGGCGGCGGCCATGGCGCGGATAAGGTCGGGACGAGGGAGTAGT</td></tr>
<tr><td rowspan="2">B</td><td rowspan="2">99</td><td>-1</td><td>89%</td><td>GGGCGGCCGTGGACGGAGAGGTGC-|CGACGGCGGCGGCCATGGCGCGGATAAGGTCGGGACGAGGGAGTAGTTGC</td></tr>
<tr><td>+2</td><td>10%</td><td>GGGCGGCCGTGGACGGAGAGGTGCG|nnCGACGGCGGCGGCCATGGCGCGGATAAGGTCGGGACGAGGGAGTAGTT</td></tr>
<tr><td rowspan="10">3</td><td rowspan="4">1</td><td rowspan="2">A</td><td rowspan="2">10</td><td>-1</td><td>10%</td><td>GGGCGGCCGTGGACGGAGAGGTGC-|CGACGGCGGCGGCGGCCATGGCGCGGATAAGGTCGGGACGAGGGAGTAGT</td></tr>
<tr><td>0</td><td>89%</td><td>GGGCGGCCGTGGACGGAGAGGTGCG|CGACGGCGGCGGCGGCCATGGCGCGGATAAGGTCGGGACGAGGGAGTAGT</td></tr>
<tr><td rowspan="2">B</td><td rowspan="2">39</td><td>0</td><td>56%</td><td>GGGCGGCCGTGGACGGAGAGGTGCG|CGACGGCGGCGGCCATGGCGCGGATAAGGTCGGGACGAGGGAGTAGTTGC</td></tr>
<tr><td>+1</td><td>39%</td><td>GGGCGGCCGTGGACGGAGAGGTGCG|nCGACGGCGGCGGCCATGGCGCGGATAAGGTCGGGACGAGGGAGTAGTTG</td></tr>
<tr><td rowspan="4">2</td><td rowspan="2">A</td><td rowspan="2">42</td><td>-1</td><td>42%</td><td>GGGCGGCCGTGGACGGAGAGGTGC-|CGACGGCGGCGGCGGCCATGGCGCGGATAAGGTCGGGACGAGGGAGTAGT</td></tr>
<tr><td>0</td><td>55%</td><td>GGGCGGCCGTGGACGGAGAGGTGCG|CGACGGCGGCGGCGGCCATGGCGCGGATAAGGTCGGGACGAGGGAGTAGT</td></tr>
<tr><td rowspan="2">B</td><td rowspan="2">16</td><td>-1</td><td>16%</td><td>GGGCGGCCGTGGACGGAGAGGTGC-|CGACGGCGGCGGCCATGGCGCGGATAAGGTCGGGACGAGGGAGTAGTTGC</td></tr>
<tr><td>0</td><td>73%</td><td>GGGCGGCCGTGGACGGAGAGGTGCG|CGACGGCGGCGGCCATGGCGCGGATAAGGTCGGGACGAGGGAGTAGTTGC</td></tr>
<tr><td rowspan="2">3</td><td>A</td><td>0</td><td>0</td><td>99%</td><td>GGGCGGCCGTGGACGGAGAGGTGCG|CGACGGCGGCGGCGGCCATGGCGCGGATAAGGTCGGGACGAGGGAGTAGT</td></tr>
<tr><td>B</td><td>87</td><td>-2</td><td>87%</td><td>GGGCGGCCGTGGACGGAGAGGTGCG|--ACGGCGGCGGCCATGGCGCGGATAAGGTCGGGACGAGGGAGTAGTTGC</td></tr>
</table>

Table S3 Data generated by the ICE software based on Sanger sequencing of regenerated plants. (Continued)

<table>
<tr><th>Line</th><th>Plant</th><th>Allele</th><th>KO-score</th><th>Indel (bp) †</th><th>Indel ratio</th><th>Sequence ‡</th></tr>
<tr><td rowspan="8">5</td><td rowspan="2">1</td><td>A</td><td>92</td><td>-7</td><td>92%</td><td>GGGCGGCCGTGGACGGAG-------|CGACGGCGGCGGCGGCCATGGCGCGGATAAGGTCGGGACGAGGGAGTAGT</td></tr>
<tr><td>B</td><td>99</td><td>+1</td><td>99%</td><td>GGGCGGCCGTGGACGGAGAGGTGCG|nCGACGGCGGCGGCCATGGCGCGGATAAGGTCGGGACGAGGGAGTAGTTG</td></tr>
<tr><td rowspan="4">2</td><td rowspan="3">A</td><td rowspan="3">70</td><td>-2</td><td>53%</td><td>GGGCGGCCGTGGACGGAGAGGTGCG|--ACGGCGGCGGCGGCCATGGCGCGGATAAGGTCGGGACGAGGGAGTAGT</td></tr>
<tr><td>-1</td><td>17%</td><td>GGGCGGCCGTGGACGGAGAGGTGC-|CGACGGCGGCGGCGGCCATGGCGCGGATAAGGTCGGGACGAGGGAGTAGT</td></tr>
<tr><td>0</td><td>21%</td><td>GGGCGGCCGTGGACGGAGAGGTGCG|CGACGGCGGCGGCGGCCATGGCGCGGATAAGGTCGGGACGAGGGAGTAGT</td></tr>
<tr><td>B</td><td>96</td><td>+1</td><td>96%</td><td>GGGCGGCCGTGGACGGAGAGGTGCG|nCGACGGCGGCGGCCATGGCGCGGATAAGGTCGGGACGAGGGAGTAGTTG</td></tr>
<tr><td rowspan="2">3</td><td>A</td><td>99</td><td>-1</td><td>99%</td><td>GGGCGGCCGTGGACGGAGAGGTGC-|CGACGGCGGCGGCGGCCATGGCGCGGATAAGGTCGGGACGAGGGAGTAGT</td></tr>
<tr><td>B</td><td>80</td><td>+2</td><td>80%</td><td>GGGCGGCCGTGGACGGAGAGGTGCG|nnCGACGGCGGCGGCCATGGCGCGGATAAGGTCGGGACGAGGGAGTAGTT</td></tr>
<tr><td rowspan="8">6</td><td rowspan="2">1</td><td>A</td><td>0</td><td>0</td><td>99%</td><td>GGGCGGCCGTGGACGGAGAGGTGCG|CGACGGCGGCGGCGGCCATGGCGCGGATAAGGTCGGGACGAGGGAGTAGT</td></tr>
<tr><td>B</td><td>99</td><td>+1</td><td>99%</td><td>GGGCGGCCGTGGACGGAGAGGTGCG|nCGACGGCGGCGGCCATGGCGCGGATAAGGTCGGGACGAGGGAGTAGTTG</td></tr>
<tr><td rowspan="3">2</td><td rowspan="2">A</td><td rowspan="2">16</td><td>-2</td><td>16%</td><td>GGGCGGCCGTGGACGGAGAGGTGCG|--ACGGCGGCGGCGGCCATGGCGCGGATAAGGTCGGGACGAGGGAGTAGT</td></tr>
<tr><td>0</td><td>82%</td><td>GGGCGGCCGTGGACGGAGAGGTGCG|CGACGGCGGCGGCGGCCATGGCGCGGATAAGGTCGGGACGAGGGAGTAGT</td></tr>
<tr><td>B</td><td>98</td><td>+1</td><td>98%</td><td>GGGCGGCCGTGGACGGAGAGGTGCG|nCGACGGCGGCGGCCATGGCGCGGATAAGGTCGGGACGAGGGAGTAGTTG</td></tr>
<tr><td rowspan="3">3</td><td rowspan="2">A</td><td rowspan="2">40</td><td>-2</td><td>40%</td><td>GGGCGGCCGTGGACGGAGAGGTGCG|--ACGGCGGCGGCGGCCATGGCGCGGATAAGGTCGGGACGAGGGAGTAGT</td></tr>
<tr><td>0</td><td>49%</td><td>GGGCGGCCGTGGACGGAGAGGTGCG|CGACGGCGGCGGCGGCCATGGCGCGGATAAGGTCGGGACGAGGGAGTAGT</td></tr>
<tr><td>B</td><td>98</td><td>+1</td><td>98%</td><td>GGGCGGCCGTGGACGGAGAGGTGCG|nCGACGGCGGCGGCCATGGCGCGGATAAGGTCGGGACGAGGGAGTAGTTG</td></tr>
<tr><td rowspan="11">8</td><td rowspan="4">1</td><td rowspan="2">A</td><td rowspan="2">35</td><td>-1</td><td>35%</td><td>GGGCGGCCGTGGACGGAGAGGTGC-|CGACGGCGGCGGCGGCCATGGCGCGGATAAGGTCGGGACGAGGGAGTAGT</td></tr>
<tr><td>0</td><td>62%</td><td>GGGCGGCCGTGGACGGAGAGGTGCG|CGACGGCGGCGGCGGCCATGGCGCGGATAAGGTCGGGACGAGGGAGTAGT</td></tr>
<tr><td rowspan="2">B</td><td rowspan="2">10</td><td>-1</td><td>10%</td><td>GGGCGGCCGTGGACGGAGAGGTGC-|CGACGGCGGCGGCCATGGCGCGGATAAGGTCGGGACGAGGGAGTAGTTGC</td></tr>
<tr><td>0</td><td>79%</td><td>GGGCGGCCGTGGACGGAGAGGTGCG|CGACGGCGGCGGCCATGGCGCGGATAAGGTCGGGACGAGGGAGTAGTTGC</td></tr>
<tr><td rowspan="7">2</td><td rowspan="4">A</td><td rowspan="4">79</td><td>-2</td><td>48%</td><td>GGGCGGCCGTGGACGGAGAGGTGCG|--ACGGCGGCGGCGGCCATGGCGCGGATAAGGTCGGGACGAGGGAGTAGT</td></tr>
<tr><td>-1</td><td>14%</td><td>GGGCGGCCGTGGACGGAGAGGTGCG|-GACGGCGGCGGCGGCCATGGCGCGGATAAGGTCGGGACGAGGGAGTAGT</td></tr>
<tr><td>0</td><td>16%</td><td>GGGCGGCCGTGGACGGAGAGGTGCG|CGACGGCGGCGGCGGCCATGGCGCGGATAAGGTCGGGACGAGGGAGTAGT</td></tr>
<tr><td>+1</td><td>17%</td><td>GGGCGGCCGTGGACGGAGAGGTGCG|nCGACGGCGGCGGCGGCCATGGCGCGGATAAGGTCGGGACGAGGGAGTAG</td></tr>
<tr><td rowspan="3">B</td><td rowspan="3">89</td><td>-2</td><td>22%</td><td>GGGCGGCCGTGGACGGAGAGGTGCG|--ACGGCGGCGGCCATGGCGCGGATAAGGTCGGGACGAGGGAGTAGTTGC</td></tr>
<tr><td>-1</td><td>33%</td><td>GGGCGGCCGTGGACGGAGAGGTGC-|CGACGGCGGCGGCCATGGCGCGGATAAGGTCGGGACGAGGGAGTAGTTGC</td></tr>
<tr><td>+1</td><td>34%</td><td>GGGCGGCCGTGGACGGAGAGGTGCG|nCGACGGCGGCGGCCATGGCGCGGATAAGGTCGGGACGAGGGAGTAGTTG</td></tr>
</table>

Table S3 Data generated by the ICE software based on Sanger sequencing of regenerated plants. (Continued)

<table>
<tr><th>Line</th><th>Plant</th><th>Allele</th><th>KO-score</th><th>Indel (bp) †</th><th>Indel ratio</th><th>Sequence ‡</th></tr>
<tr><td rowspan="5">8</td><td rowspan="5">3</td><td rowspan="2">A</td><td rowspan="2">19</td><td>0</td><td>59%</td><td>GGGCGGCCGTGGACGGAGAGGTGCG|CGACGGCGGCGGCGGCCATGGCGCGGATAAGGTCGGGACGAGGGAGTAGT</td></tr>
<tr><td>+1</td><td>19%</td><td>GGGCGGCCGTGGACGGAGAGGTGCG|nCGACGGCGGCGGCGGCCATGGCGCGGATAAGGTCGGGACGAGGGAGTAG</td></tr>
<tr><td rowspan="3">B</td><td rowspan="3">53</td><td>-1</td><td>40%</td><td>GGGCGGCCGTGGACGGAGAGGTGC-|CGACGGCGGCGGCCATGGCGCGGATAAGGTCGGGACGAGGGAGTAGTTGC</td></tr>
<tr><td>0</td><td>38%</td><td>GGGCGGCCGTGGACGGAGAGGTGCG|CGACGGCGGCGGCCATGGCGCGGATAAGGTCGGGACGAGGGAGTAGTTGC</td></tr>
<tr><td>+1</td><td>13%</td><td>GGGCGGCCGTGGACGGAGAGGTGCG|nCGACGGCGGCGGCCATGGCGCGGATAAGGTCGGGACGAGGGAGTAGTTG</td></tr>
<tr><td rowspan="9">10</td><td rowspan="3">1</td><td rowspan="2">A</td><td rowspan="2">29</td><td>-2</td><td>29%</td><td>GGGCGGCCGTGGACGGAGAGGTGCG|--ACGGCGGCGGCGGCCATGGCGCGGATAAGGTCGGGACGAGGGAGTAGT</td></tr>
<tr><td>0</td><td>59%</td><td>GGGCGGCCGTGGACGGAGAGGTGCG|CGACGGCGGCGGCGGCCATGGCGCGGATAAGGTCGGGACGAGGGAGTAGT</td></tr>
<tr><td>B</td><td>98</td><td>+1</td><td>98%</td><td>GGGCGGCCGTGGACGGAGAGGTGCG|nCGACGGCGGCGGCCATGGCGCGGATAAGGTCGGGACGAGGGAGTAGTTG</td></tr>
<tr><td rowspan="3">2</td><td>A</td><td>99</td><td>-2</td><td>99%</td><td>GGGCGGCCGTGGACGGAGAGGTGCG|--ACGGCGGCGGCGGCCATGGCGCGGATAAGGTCGGGACGAGGGAGTAGT</td></tr>
<tr><td rowspan="2">B</td><td rowspan="2">94</td><td>-1</td><td>49%</td><td>GGGCGGCCGTGGACGGAGAGGTGC-|CGACGGCGGCGGCCATGGCGCGGATAAGGTCGGGACGAGGGAGTAGTTGC</td></tr>
<tr><td>+1</td><td>45%</td><td>GGGCGGCCGTGGACGGAGAGGTGCG|nCGACGGCGGCGGCCATGGCGCGGATAAGGTCGGGACGAGGGAGTAGTTG</td></tr>
<tr><td rowspan="3">3</td><td rowspan="2">A</td><td rowspan="2">32</td><td>-1</td><td>32%</td><td>GGGCGGCCGTGGACGGAGAGGTGC-|CGACGGCGGCGGCGGCCATGGCGCGGATAAGGTCGGGACGAGGGAGTAGT</td></tr>
<tr><td>0</td><td>65%</td><td>GGGCGGCCGTGGACGGAGAGGTGCG|CGACGGCGGCGGCGGCCATGGCGCGGATAAGGTCGGGACGAGGGAGTAGT</td></tr>
<tr><td>B</td><td>98</td><td>+1</td><td>98%</td><td>GGGCGGCCGTGGACGGAGAGGTGCG|nCGACGGCGGCGGCCATGGCGCGGATAAGGTCGGGACGAGGGAGTAGTTG</td></tr>
<tr><td rowspan="8">11</td><td rowspan="4">1</td><td rowspan="2">A</td><td rowspan="2">25</td><td>0</td><td>70%</td><td>GGGCGGCCGTGGACGGAGAGGTGCG|CGACGGCGGCGGCGGCCATGGCGCGGATAAGGTCGGGACGAGGGAGTAGT</td></tr>
<tr><td>-2</td><td>25%</td><td>GGGCGGCCGTGGACGGAGAGGTGCG|--ACGGCGGCGGCGGCCATGGCGCGGATAAGGTCGGGACGAGGGAGTAGT</td></tr>
<tr><td rowspan="2">B</td><td rowspan="2">86</td><td>+1</td><td>86%</td><td>GGGCGGCCGTGGACGGAGAGGTGCG|nCGACGGCGGCGGCCATGGCGCGGATAAGGTCGGGACGAGGGAGTAGTTG</td></tr>
<tr><td>+3</td><td>11%</td><td>GGGCGGCCGTGGACGGAGAGGTGCG|nnnCGACGGCGGCGGCCATGGCGCGGATAAGGTCGGGACGAGGGAGTAGT</td></tr>
<tr><td rowspan="2">2</td><td>A</td><td>0</td><td>0</td><td>92%</td><td>GGGCGGCCGTGGACGGAGAGGTGCG|CGACGGCGGCGGCGGCCATGGCGCGGATAAGGTCGGGACGAGGGAGTAGT</td></tr>
<tr><td>B</td><td>99</td><td>+1</td><td>99%</td><td>GGGCGGCCGTGGACGGAGAGGTGCG|nCGACGGCGGCGGCCATGGCGCGGATAAGGTCGGGACGAGGGAGTAGTTG</td></tr>
<tr><td rowspan="2">3</td><td>A</td><td>0</td><td>0</td><td>91%</td><td>GGGCGGCCGTGGACGGAGAGGTGCG|CGACGGCGGCGGCGGCCATGGCGCGGATAAGGTCGGGACGAGGGAGTAGT</td></tr>
<tr><td>B</td><td>98</td><td>+1</td><td>98%</td><td>GGGCGGCCGTGGACGGAGAGGTGCG|nCGACGGCGGCGGCCATGGCGCGGATAAGGTCGGGACGAGGGAGTAGTTG</td></tr>
<tr><td rowspan="3">16</td><td rowspan="3">1</td><td rowspan="2">A</td><td rowspan="2">11</td><td>-3</td><td>67%</td><td>GGGCGGCCGTGGACGGAGAGGT---|CGACGGCGGCGGCGGCCATGGCGCGGATAAGGTCGGGACGAGGGAGTAGT</td></tr>
<tr><td>-2</td><td>11%</td><td>GGGCGGCCGTGGACGGAGAGGTGC-|-GACGGCGGCGGCGGCCATGGCGCGGATAAGGTCGGGACGAGGGAGTAGT</td></tr>
<tr><td>B</td><td>95</td><td>+1</td><td>95%</td><td>GGGCGGCCGTGGACGGAGAGGTGCG|nCGACGGCGGCGGCCATGGCGCGGATAAGGTCGGGACGAGGGAGTAGTTG</td></tr>
</table>

Table S3 Data generated by the ICE software based on Sanger sequencing of regenerated plants. (Continued)

<table>
<tr><th>Line</th><th>Plant</th><th>Allele</th><th>KO-score</th><th>Indel (bp) †</th><th>Indel ratio</th><th>Sequence ‡</th></tr>
<tr><td rowspan="6">16</td><td rowspan="4">2</td><td rowspan="3">A</td><td rowspan="3">0</td><td>-3</td><td>19%</td><td>GGGCGGCCGTGGACGGAGAGGTGC-|--ACGGCGGCGGCGGCCATGGCGCGGATAAGGTCGGGACGAGGGAGTAGT</td></tr>
<tr><td>-3</td><td>29%</td><td>GGGCGGCCGTGGACGGAGAGGT---|CGACGGCGGCGGCGGCCATGGCGCGGATAAGGTCGGGACGAGGGAGTAGT</td></tr>
<tr><td>0</td><td>42%</td><td>GGGCGGCCGTGGACGGAGAGGTGCG|CGACGGCGGCGGCGGCCATGGCGCGGATAAGGTCGGGACGAGGGAGTAGT</td></tr>
<tr><td>B</td><td>96</td><td>+1</td><td>96%</td><td>GGGCGGCCGTGGACGGAGAGGTGCG|nCGACGGCGGCGGCCATGGCGCGGATAAGGTCGGGACGAGGGAGTAGTTG</td></tr>
<tr><td rowspan="2">3</td><td>A</td><td>97</td><td>-1</td><td>97%</td><td>GGGCGGCCGTGGACGGAGAGGTGC-|CGACGGCGGCGGCGGCCATGGCGCGGATAAGGTCGGGACGAGGGAGTAGT</td></tr>
<tr><td>B</td><td>99</td><td>+1</td><td>99%</td><td>GGGCGGCCGTGGACGGAGAGGTGCG|nCGACGGCGGCGGCCATGGCGCGGATAAGGTCGGGACGAGGGAGTAGTTG</td></tr>
<tr><td rowspan="8">19</td><td rowspan="2">1</td><td>A</td><td>99</td><td>-1</td><td>99%</td><td>GGGCGGCCGTGGACGGAGAGGTGC-|CGACGGCGGCGGCGGCCATGGCGCGGATAAGGTCGGGACGAGGGAGTAGT</td></tr>
<tr><td>B</td><td>94</td><td>-1</td><td>94%</td><td>GGGCGGCCGTGGACGGAGAGGTGC-|CGACGGCGGCGGCCATGGCGCGGATAAGGTCGGGACGAGGGAGTAGTTGC</td></tr>
<tr><td rowspan="4">2</td><td rowspan="3">A</td><td rowspan="3">74</td><td>-1</td><td>74%</td><td>GGGCGGCCGTGGACGGAGAGGTGC-|CGACGGCGGCGGCGGCCATGGCGCGGATAAGGTCGGGACGAGGGAGTAGT</td></tr>
<tr><td>-3</td><td>10%</td><td>GGGCGGCCGTGGACGGAGAGGTGC-|--ACGGCGGCGGCGGCCATGGCGCGGATAAGGTCGGGACGAGGGAGTAGT</td></tr>
<tr><td>-3</td><td>12%</td><td>GGGCGGCCGTGGACGGAGAGGT---|CGACGGCGGCGGCGGCCATGGCGCGGATAAGGTCGGGACGAGGGAGTAGT</td></tr>
<tr><td>B</td><td>100</td><td>-8</td><td>100%</td><td>GGGCGGCCGTGGACGGAGAGGTGCG|--------GCGGCCATGGCGCGGATAAGGTCGGGACGAGGGAGTAGTTGC</td></tr>
<tr><td rowspan="2">3</td><td>A</td><td>0</td><td>-3</td><td>90%</td><td>GGGCGGCCGTGGACGGAGAGGT---|CGACGGCGGCGGCGGCCATGGCGCGGATAAGGTCGGGACGAGGGAGTAGT</td></tr>
<tr><td>B</td><td>100</td><td>-8</td><td>100%</td><td>GGGCGGCCGTGGACGGAGAGGTGCG|--------GCGGCCATGGCGCGGATAAGGTCGGGACGAGGGAGTAGTTGC</td></tr>
<tr><td rowspan="9">20</td><td rowspan="3">1</td><td rowspan="2">A</td><td rowspan="2">43</td><td>-2</td><td>30%</td><td>GGGCGGCCGTGGACGGAGAGGTGCG|--ACGGCGGCGGCGGCCATGGCGCGGATAAGGTCGGGACGAGGGAGTAGT</td></tr>
<tr><td>0</td><td>53%</td><td>GGGCGGCCGTGGACGGAGAGGTGCG|CGACGGCGGCGGCGGCCATGGCGCGGATAAGGTCGGGACGAGGGAGTAGT</td></tr>
<tr><td>B</td><td>99</td><td>+1</td><td>99%</td><td>GGGCGGCCGTGGACGGAGAGGTGCG|nCGACGGCGGCGGCCATGGCGCGGATAAGGTCGGGACGAGGGAGTAGTTG</td></tr>
<tr><td rowspan="3">2</td><td rowspan="2">A</td><td rowspan="2">24</td><td>-1</td><td>24%</td><td>GGGCGGCCGTGGACGGAGAGGTGC-|CGACGGCGGCGGCGGCCATGGCGCGGATAAGGTCGGGACGAGGGAGTAGT</td></tr>
<tr><td>0</td><td>74%</td><td>GGGCGGCCGTGGACGGAGAGGTGCG|CGACGGCGGCGGCGGCCATGGCGCGGATAAGGTCGGGACGAGGGAGTAGT</td></tr>
<tr><td>B</td><td>98</td><td>+1</td><td>98%</td><td>GGGCGGCCGTGGACGGAGAGGTGCG|nCGACGGCGGCGGCCATGGCGCGGATAAGGTCGGGACGAGGGAGTAGTTG</td></tr>
<tr><td rowspan="3">3</td><td rowspan="2">A</td><td rowspan="2">29</td><td>-2</td><td>24%</td><td>GGGCGGCCGTGGACGGAGAGGTGCG|--ACGGCGGCGGCGGCCATGGCGCGGATAAGGTCGGGACGAGGGAGTAGT</td></tr>
<tr><td>0</td><td>63%</td><td>GGGCGGCCGTGGACGGAGAGGTGCG|CGACGGCGGCGGCGGCCATGGCGCGGATAAGGTCGGGACGAGGGAGTAGT</td></tr>
<tr><td>B</td><td>98</td><td>+1</td><td>98%</td><td>GGGCGGCCGTGGACGGAGAGGTGCG|nCGACGGCGGCGGCCATGGCGCGGATAAGGTCGGGACGAGGGAGTAGTTG</td></tr>
</table>

† Indels indicate -; deletion, +; insertion and the number of nucleotides.

‡ Dashes represent deletions, and N represents insertion at the guide RNA region. Vertical line represents the expected cut site.
